# The Australian Reproductive Genetic Carrier Screening Project (Mackenzie’s Mission): Design and Implementation

**DOI:** 10.3390/jpm12111781

**Published:** 2022-10-28

**Authors:** Alison D. Archibald, Belinda J. McClaren, Jade Caruana, Erin Tutty, Emily A. King, Jane L. Halliday, Stephanie Best, Anaita Kanga-Parabia, Bruce H. Bennetts, Corrina C. Cliffe, Evanthia O. Madelli, Gladys Ho, Jan Liebelt, Janet C. Long, Jeffrey Braithwaite, Jillian Kennedy, John Massie, Jon D. Emery, Julie McGaughran, Justine E. Marum, Kirsten Boggs, Kristine Barlow-Stewart, Leslie Burnett, Lisa Dive, Lucinda Freeman, Mark R. Davis, Martin J. Downes, Mathew Wallis, Monica M. Ferrie, Nicholas Pachter, Paul A. Scuffham, Rachael Casella, Richard J. N. Allcock, Royston Ong, Samantha Edwards, Sarah Righetti, Sebastian Lunke, Sharon Lewis, Susan P. Walker, Tiffany F. Boughtwood, Tristan Hardy, Ainsley J. Newson, Edwin P. Kirk, Nigel G. Laing, Martin B. Delatycki, The Mackenzie’s Mission Study Team

**Affiliations:** 1 Victorian Clinical Genetics Services, Parkville, VIC 3052, Australia; 2 Murdoch Children’s Research Institute, Parkville, VIC 3052, Australia; 3 Department of Paediatrics, University of Melbourne, Parkville, VIC 3052, Australia; 4 Australian Genomics, Parkville, VIC 3052, Australia; 5 Bruce Lefroy Centre, Murdoch Children’s Research Institute, Parkville, VIC 3052, Australia; 6 Peter MacCallum Cancer Centre, Melbourne, VIC 3000, Australia; 7 Victorian Comprehensive Cancer Centre, Melbourne, VIC 3000, Australia; 8 Sir Peter MacCallum Department of Oncology, University of Melbourne, Melbourne, VIC 3000, Australia; 9 Sydney Genome Diagnostics, The Children’s Hospital at Westmead, Westmead, NSW 2145, Australia; 10 Specialty of Genomic Medicine, The Children’s Hospital at Westmead Clinical School, Faculty of Medicine and Health, University of Sydney, Westmead, NSW 2145, Australia; 11 NSW Health Pathology Randwick Genomics Laboratory, Randwick, NSW 2031, Australia; 12 South Australian Clinical Genetics Service, North Adelaide, SA 5006, Australia; 13 Women’s and Children’s Hospital, North Adelaide, SA 5006, Australia; 14 Repromed, Dulwich, SA 5065, Australia; 15 Australian Institute of Health Innovation, Macquarie University, North Ryde, NSW 2109, Australia; 16 International Society for Quality in Health Care, D02 YY23 Dublin, Ireland; 17 Genetic Services of Western Australia, Subiaco, WA 6008, Australia; 18 Department of Respiratory Medicine, The Royal Children’s Hospital, Parkville, VIC 3052, Australia; 19 Department of General Practice and Centre for Cancer Research, University of Melbourne, Melbourne, VIC 3000, Australia; 20 Genetic Health Queensland, Royal Brisbane and Women’s Hospital, Herston, QLD 4006, Australia; 21 School of Medicine, University of Queensland, St Lucia, QLD 4072, Australia; 22 Centre for Clinical Genetics, Sydney Children’s Hospital, Randwick, NSW 2031, Australia; 23 Department of Clinical Genetics, The Children’s Hospital at Westmead, Westmead, NSW 2145, Australia; 24 Northern Clinical School, Faculty of Medicine and Health, University of Sydney, St Leonards, NSW 2065, Australia; 25 Faculty of Medicine and Health, University of New South Wales, Sydney, NSW 2052, Australia; 26 Garvan Institute of Medical Research, Darlinghurst, NSW 2010, Australia; 27 St Vincent’s Clinical School, University of New South Wales, Darlinghurst, NSW 2010, Australia; 28 Invitae Australia, Alexandria, NSW 2015, Australia; 29 Graduate School of Health, University of Technology Sydney, Ultimo, NSW 2007, Australia; 30 Sydney Health Ethics, Sydney School of Public Health, Faculty of Medicine and Health, University of Sydney, Camperdown, NSW 2006, Australia; 31 School of Women’s and Children’s Health, University of New South Wales, Randwick, NSW 2031, Australia; 32 Department of Diagnostic Genomics, PathWest Laboratory Medicine, Nedlands, WA 6009, Australia; 33 Centre for Medical Research, University of Western Australia, Nedlands, WA 6009, Australia; 34 Menzies Health Institute Queensland, Griffith University, Gold Coast, QLD 4222, Australia; 35 Centre for Applied Health Economics, School of Medicine and Dentistry, Griffith University, Nathan, QLD 4111, Australia; 36 Tasmanian Clinical Genetics Service, Tasmanian Health Service, Hobart, TAS 7000, Australia; 37 School of Medicine and Menzies Institute for Medical Research, University of Tasmania, Hobart, TAS 7000, Australia; 38 Genetic Support Network of Victoria, Parkville, VIC 3052, Australia; 39 King Edward Memorial Hospital, Subiaco, WA 6008, Australia; 40 Faculty of Health and Medical Sciences, University of Western Australia, Perth, WA 6009, Australia; 41 Independent Researcher; 42 School of Biomedical Sciences, University of Western Australia, Perth, WA 6009, Australia; 43 Harry Perkins Institute of Medical Research, Nedlands, WA 6009, Australia; 44 Department of Pathology, University of Melbourne, Melbourne, VIC 3000, Australia; 45 Mercy Perinatal, Mercy Hospital for Women, Heidelberg, VIC 3084, Australia; 46 Department of Obstetrics and Gynaecology, University of Melbourne, Parkville, VIC 3010, Australia; 47 Monash IVF Group, Richmond, VIC 3121, Australia; 48 SA Pathology, Adelaide, SA 5000, Australia

**Keywords:** reproductive genetic carrier screening, implementation science, bioethics, health economics, psychosocial outcomes

## Abstract

Reproductive genetic carrier screening (RGCS) provides people with information about their chance of having children with autosomal recessive or X-linked genetic conditions, enabling informed reproductive decision-making. RGCS is recommended to be offered to all couples during preconception or in early pregnancy. However, cost and a lack of awareness may prevent access. To address this, the Australian Government funded Mackenzie’s Mission—the Australian Reproductive Genetic Carrier Screening Project. Mackenzie’s Mission aims to assess the acceptability and feasibility of an easily accessible RGCS program, provided free of charge to the participant. In study Phase 1, implementation needs were mapped, and key study elements were developed. In Phase 2, RGCS is being offered by healthcare providers educated by the study team. Reproductive couples who provide consent are screened for over 1200 genes associated with >750 serious, childhood-onset genetic conditions. Those with an increased chance result are provided comprehensive genetic counseling support. Reproductive couples, recruiting healthcare providers, and study team members are also invited to complete surveys and/or interviews. In Phase 3, a mixed-methods analysis will be undertaken to assess the program outcomes, psychosocial implications and implementation considerations alongside an ongoing bioethical analysis and a health economic evaluation. Findings will inform the implementation of an ethically robust RGCS program.

## 1. Background

Reproductive genetic carrier screening (RGCS) is a form of genetic testing that provides prospective parents with information about their chance of having children with severe autosomal recessive and X-linked genetic conditions, thus promoting choice and reproductive autonomy [1,2]. While individually rare, it is estimated that 1–2% of reproductive couples have an increased chance of having children impacted by such conditions [3,4]. Such couples (henceforth referred to as ‘increased chance couples’) may choose to access reproductive options either to avoid, or to plan and prepare for the birth of children with the condition in accordance with their values [5]. These options include prenatal diagnosis (PND), in vitro fertilization (IVF) with preimplantation genetic testing for monogenic disorders (PGT-M), using donor gametes, adoption/fostering or not having children. 

Families can comprise a broad range of structures, and parents may or may not have genetic links with their child (for example, if gamete or embryo donors are used). With respect to RGCS, there are two ‘genetic parents’ (of male and female sex) for the prospective or current pregnancy who can be considered the ‘reproductive couple’. It is important to acknowledge non-genetic parents who may also be involved in the RGCS process with respect to pre-test deliberation and reproductive decision-making. Thus, when referring to ‘couples’ engaging in RGCS, we refer to the collective group who will use the information gained from RGCS to inform future reproductive choices. 

In the past, RGCS was primarily offered to those from certain ethnic backgrounds with a high disease prevalence. However, the recessive nature of these conditions, barriers in family communication of genetic information, and delayed or underdiagnosis of genetic conditions mean most affected children are born to parents who have no known prior history of the condition [6]. Additionally, as populations become increasingly multiethnic, relying on ethnicity ceases to be an effective way to guide offers of RGCS [7,8]. Numerous peak bodies now recognize RGCS as relevant to all people planning a pregnancy or in early pregnancy regardless of family history or ethnicity [9,10,11].

Research demonstrates there is interest in and support for RGCS, provided it is optional and those offered screening are given sufficient time and information to consider their decision [9,12,13,14,15]. Research has shown increased chance couples may initially express shock and anxiety [16], however, over time report feeling empowered by the information and the ability to make value-congruent reproductive choices [17,18]. These findings highlight the importance of providing RGCS within a model that incorporates informed decision-making with high-quality pre-test information and the provision of appropriate genetic counseling support [18,19].

Internationally, there is substantial variation in access to RGCS, as well as differences in the number and type of conditions screened, how results are returned and managed, if the reproductive couple is screened sequentially or simultaneously, and whether government-funded RGCS is available [1,2]. In Australia, RGCS is currently available largely on a user-pays basis, through commercial entities. However, the Australian Federal Government’s Department of Health recently announced a plan for government-funded carrier screening for three conditions (cystic fibrosis, spinal muscular atrophy and fragile X syndrome) to be implemented in late 2023. 

RGCS is usually ordered through healthcare providers, such as general practitioners, obstetricians, midwives, fertility specialists and genetic health professionals. Barriers to RGCS, including test costs and a lack of awareness amongst healthcare providers and the general community, mean that most increased chance couples are not given the opportunity to learn this information prior to the birth of an affected child [20]. Currently, most people who access RGCS do so in early pregnancy, despite this information being most useful prior to conception [20]. Those accessing RGCS also tend to live in metropolitan areas and utilize private healthcare [20,21,22]. Given this inequality in access, a population-based screening approach would enable RGCS to be made more widely available. 

In 2017, Mackenzie Casella, the daughter of Rachael and Jonathan Casella, died at 7 months of age from spinal muscular atrophy, an autosomal recessive genetic condition for which her parents were unknowingly genetic carriers. The Casellas had not been made aware that RGCS for spinal muscular atrophy was possible and available, before or during their pregnancy. Since then, the Casellas have been vocal advocates for RGCS and, along with patient support organizations, healthcare practitioners, genetic health professionals and relevant peak bodies, have lobbied the Australian Government for funded RGCS. In 2018, it was announced the Government would fund a large national research study named after Mackenzie [23,24]. Here, we describe the design of this study: Mackenzie’s Mission (MM)—the Australian Reproductive Genetic Carrier Screening Project. 

## 2. Methods

### 2.1. Funding and Ethics Committee Approval

This study was funded by the Australian Government’s Medical Research Future Fund as part of the Genomics Health Futures Mission. Human Research Ethics Committee (HREC) approval was obtained to conduct the study in all Australian states and territories (see Supplementary File S1 for a list of HREC approvals). Site-specific governance approval was obtained for recruitment through 22 publicly funded healthcare services (hospitals and clinical genetics services). Site-specific governance approval was not required for recruitment in private healthcare settings (general practice, private obstetrics, midwifery and ultrasound, and some fertility services) as HREC approval was deemed to be sufficient. In addition to the incidental recruitment of Aboriginal and Torres Strait Islander participants, specific HREC approval was obtained to purposefully recruit in Indigenous healthcare settings. 

### 2.2. Aims

The primary aim of MM is to develop and implement a free, easily accessible RGCS program, and assess its feasibility and acceptability. Objectives include:

- to assess program outcomes, including, the offer and uptake of RGCS, frequency of increased chance couples, and their reproductive decisions and outcomes;
- to understand the screening experience by measuring psychosocial impacts, including anxiety, factors influencing decision-making, decisional conflict and regret;
- to evaluate the implementation of RGCS by identifying possible barriers and enablers for key stakeholders, including people offered screening, healthcare providers (HCPs), and clinical and laboratory services delivering RGCS;
- to assess the health economic implications of RGCS;
- to understand the bioethical aspects of RGCS and explore the ethical issues arising during the implementation and evaluation of the program.

### 2.3. Study Governance

**Figure 1:**
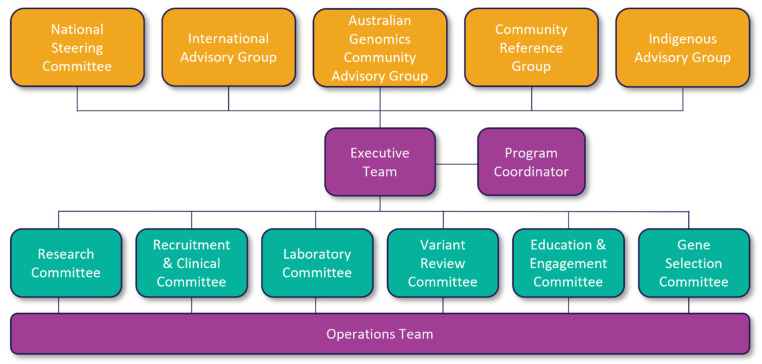
Mackenzie’s Mission’s governance structure.

The MM governance structure is summarized in Figure 1. The study is administered by Australian Genomics [25], a national research network that has a charter to build evidence for the integration of genomic medicine into the healthcare system. 

MM has a National Steering Committee and an International Advisory Group who advise on the strategic direction, research activities and implementation of the study. An Indigenous Advisory Group was convened to advise on the development of a culturally appropriate recruitment approach and materials for Aboriginal and Torres Strait Islander participants. The study also has input from the Australian Genomics Community Advisory Group and established its own Community Reference Group, who have provided input into the design and language of the participant materials and advised on accessibility. 

Six project committees were established to oversee key aspects of the study: Gene Selection, Laboratory, Variant Review, Education and Engagement, Research, and Recruitment and Clinical. The membership of these committees includes representatives from all Australian states and territories, professional bodies including the Royal Australian and New Zealand College of Obstetricians and Gynaecologists (RANZCOG), Royal Australian College of General Practitioners (RACGP), Human Genetics Society of Australasia (HGSA), and Royal College of Pathologists of Australasia (RCPA), as well as professionals involved in the delivery of RGCS. 

The MM study team comprises an Executive Team (the Study Leads, Program Coordinator and Managing Director of Australian Genomics) and an Operations Team (medical scientists, genetic counselors, clinical geneticists, researchers and administrative staff). 

### 2.4. Study Design

**Figure 2:**
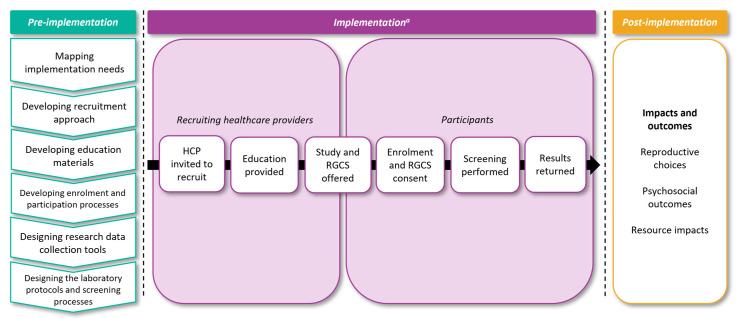
Mackenzie’s Mission’s study design showing the implementation phases.

^a^ The processes were piloted in four states/territories (Phase 2a) before national implementation (Phase 2b).

The MM study design is outlined in Figure 2. The study commenced in 2019 with an initial plan to recruit 10,000 couples and a completion date of December 2021. However, the SARS-CoV-2 pandemic impacted recruitment and laboratory testing. Thus, the study was extended with a revised recruitment target of 8350 couples and a completion date of December 2022. A longer-term follow-up of the reproductive outcomes will be undertaken in subsequent years. 

The study comprises three phases: (**1) Pre-implementation**, which involved study design and development (2019); (**2) Implementation**, which comprises the two stages of (*2a) Piloting and early implementation* (2020), and (*2b) Nation-wide implementation* (2021–2022); and (**3) Post-implementation**, assessing outcomes (2022 and beyond). The recruitment of couples was designed to occur in a staged manner, with the rate of recruitment increasing progressively over the course of the study. In Phase 2a, screening was offered in four states/territories: the Australian Capital Territory (ACT), New South Wales (NSW), Victoria (VIC) and Western Australia (WA). In Phase 2b, screening was extended to all Australian states and territories, including the Northern Territory (NT), Queensland (QLD), South Australia (SA) and Tasmania (TAS).

#### Evaluation of the Mackenzie’s Mission Reproductive Genetic Carrier Screening Program

As MM is a research study, evaluation is embedded throughout. There are four research streams taking an integrated approach to evaluating the program: psychosocial and epidemiology; bioethics; health economics; and implementation science. Within the psychosocial and epidemiology, health economics, and implementation science streams, empirical research is being conducted using a mixed-methods approach. Data sources include HCPs, people offered RGCS through MM, and the MM study team. The health economics analysis includes an assessment of the health economic impacts of RGCS using data from the literature, the MM study, and data provided by clinical and laboratory services. A bioethics critical analysis, exploring the relevant ethical issues and recommendations for how they can be addressed in the future delivery of RGCS, is being undertaken in parallel. This has focused on the ethical aspects of delivering RGCS at the population scale and has included a consideration of issues such as gene selection and reporting, ethical frameworks, eugenics, and condition severity [5,26,27,28,29,30]. Bioethical perspectives and analysis have been integrated into the consideration of ethical issues that arise throughout MM, and ethical dilemmas encountered while implementing MM have informed the focus of conceptual bioethics research. Collectively, the outcomes from each research stream will inform the development and future implementation of RGCS in Australia. 

### 2.5. Phase 1—Pre-Implementation: Study Design and Development

Figure 2 summarizes the key stages of study Phase 1. During this stage, the Indigenous and Community Reference groups were established, and began to have input into the study design and development. 

#### 2.5.1. Mapping Implementation Needs

To explore the initial HCP knowledge and perspectives on RGCS, and to identify the potential barriers and facilitators to offering RGCS to people in usual care, an HCP survey was developed (HCP survey 1) [31]. This survey used the validated Patient Safety Behaviour Survey [32] informed by the Theoretical Domains Framework, a behavior change framework comprising a compilation of both psychological and organizational theories, which influence behavior and behavioral change [33]. HCPs who may offer RGCS to people seen in their usual practice were approached opportunistically at relevant conference presentations, education seminars and professional meetings, and asked to complete the survey. 

Discussions were undertaken with clinical, laboratory and administrative staff experienced in genetic testing and screening service delivery to understand the elements required to be incorporated into the RGCS program design. Particular attention was focused on ensuring the appropriate resources and supports were in place for increased chance couples, as access can vary by geographical location and healthcare setting across Australia. Physicians specializing in conditions/groups of conditions were engaged and informed about the study in recognition that they may need to be called upon to provide increased chance couples with further information on the condition. 

#### 2.5.2. Developing the Recruitment Approach

To aid this research, an electronic study database was created and housed in the Research Electronic Data Capture (REDCap) platform hosted at the Murdoch Children’s Research Institute [34,35]. To plan for a future, widely accessible RGCS program, it was essential to capture the experience of delivering the screening in a variety of settings. In Australia, healthcare is multifaceted and provided through services that are funded publicly and/or privately; thus, recruitment needed to represent all avenues through which people currently access pre- and early pregnancy healthcare in Australia. General practice, obstetrics, midwifery, ultrasound, fertility and genetics services were identified as potential healthcare settings in which recruitment could occur. Multiple strategies were used to identify recruiting clinics and HCPs (referred to as rHCPs hereafter), including through: existing networks established through their involvement in delivering RGCS or prenatal screening services; Primary Healthcare Networks (groups of independent Australian Government-funded organizations that coordinate primary healthcare in their region); practitioner education events; online searches of practitioners in particular geographic areas; posts on selected relevant social media groups; presentations at conferences; professional networks; word of mouth; and snowball sampling by rHCPs. It was considered essential to recruit as representative of a sample of the Australian population as possible to ensure wide generalizability of the research outcomes from the study. Recruitment targets were set to ensure the representation of people in a variety of geographic areas across Australia using 2016 birth rates in defined geographic regions [36]. Recruitment targets also incorporated the proportion of people accessing public and private healthcare in each region [37]. 

Previous studies show that HCPs who currently provide pre- and early pregnancy care are ideally placed to introduce the notion of RGCS, but that time, a lack of knowledge about RGCS, and competing priorities are barriers to them offering RGCS [38,39]. To address these challenges, an online portal was developed for participant enrollment, education and consent, thereby reducing the requirements of rHCPs to provide detailed information about RGCS. Eligibility criteria were designed to be deliberately broad to enable a wide range of people to take part (Table 1). An effort has been directed to ensure that the study is accessible to people from a broad spectrum of ethnicities, including Aboriginal and Torres Strait Islander people, people with limited English, those without computer/Internet access and those with disabilities. People who already know that they have an increased chance of having children with a serious genetic condition (due to family history and/or previous genetic testing) are included in the study so they can access RGCS for other conditions. 

#### 2.5.3. Developing Education Materials 

Phase 1 also involved developing education materials for rHCPs and participants. rHCP education was initially delivered by the study’s genetic counselors in each state with the use of a PowerPoint presentation either in the rHCP’s clinic or via videoconferencing. The educational content was refined over time with feedback from rHCPs. Once finalized, the PowerPoint presentation was recorded and offered as an option for rHCPs to view in their own time. An online education module was also developed for rHCPs, which comprised written information, videos, infographics and a quiz to self-assess key knowledge concepts. 

An education module for potential participants invited to take part in the study by their rHCP was developed comprising videos, infographics and knowledge questions. The education module informs participants about the study and assists them in deciding whether to have RGCS. The information conveyed outlines: Mackenzie’s story; the study aims; RGCS; the types of genetic conditions included in the screening; inheritance patterns; what it means to be a genetic carrier; how the screening process works; the limitations of the screening; and the possible outcomes of screening, including reproductive options for increased chance couples. A decision aid, which supports decision-making based on consideration of the benefits and limitations of screening with respect to the participant’s views and beliefs, was developed based on previous relevant decision aids [40,41] and is described in detail elsewhere [42]. 

#### 2.5.4. Developing the Enrollment and Participation Process

An online participant portal, including the education module and decision aid, was designed as a convenient way for couples who were invited to take part in the study by their rHCP to enroll and participate. The participant portal was designed by the digital health technology company, Curve Tomorrow, based on the design of the Australian Genomics dynamic consent platform, ‘CTRL’ [43], and with involvement from the MM study team. The participant portal is a web application accessible from smartphones, tablets and computers. Information entered into the participant portal is automatically transferred into the MM electronic study database. Each couple accesses the portal with a unique access code, linked to their rHCP. This access code is provided by the rHCP on a study invitation form (see Supplementary File S2) either in hard copy or electronically. On one side of the invitation form is a brief description of the study and instructions for how to access the participant portal, and on the reverse, is a test request form which is completed by the rHCP to be used for those who accept RGCS. 

To capture the breadth of views on RGCS, the portal was designed to allow for the participation of: those who want to have the screening, those who do not, and those who are unsure. The portal includes: a short introductory video on the study; eligibility questions; a provision of consent to participate in the research; a collection of demographic information; the education module; the optional decision aid [42]; clinical consent for RGCS; an evaluation survey; and optional dynamic consent preferences for data sharing. 

Consent for undergoing RGCS is separate from the consent to take part in the research study, so that the RGCS consent can be provided after the participant has viewed the education module and engaged with the decision aid. The RGCS consent process includes: the purpose of screening; limitations; possible outcomes; the use of information gained through the genetic carrier screening; and data sharing for research. Optional consent preferences for data sharing with other research studies were adapted from the Australian Genomics CTRL platform [43], using a dynamic approach which allows participants to revisit the participant portal and change their preferences at any time.

To maximize accessibility, modified enrollment pathways were developed to support enrollment for people conceiving using a known egg, sperm or embryo donor(s), people with limited English, Aboriginal and Torres Strait Islander people, those who do not have computer/Internet access, and people with disabilities. For people conceiving using a known donor(s) (including the ‘genetic parents’, the person/people who will be parenting/raising the child/ren and the surrogate (if surrogacy is involved)), a separate online enrollment pathway was developed to accommodate and involve the various possible family structures, using inclusive language. A separate online enrollment pathway, with content translated into Arabic and Simplified Chinese (the two most commonly spoken languages in Australia other than English), was also developed for people not fluent in English. A researcher-assisted telephone enrollment pathway was developed for people who require additional support with enrollment, including those who speak other languages and require an interpreter. Hard copy enrollment forms were developed for participants unable to access the online participant portal. To ensure the representation of Aboriginal and Torres Strait Islander people, the study team worked with specific Indigenous health services to develop a tailored recruitment approach involving visual recruitment aids, hard copy enrollment forms and a supported enrollment process. The participant portal was designed in accordance with accessibility principles and standards, to make it accessible to people with disabilities. A free-call study phone number was established and staffed during office hours by the study’s genetic counselors to respond to participants’ questions and provide support as needed. All components of the participant portal and modified enrollment pathways were reviewed by key stakeholders including the Community Reference Group. 

#### 2.5.5. Designing the Data Collection Tools

Data collection tools were developed and piloted to assess the experience and outcomes of participating rHCPs, those offered RGCS and the MM study team. 

##### Data Collection Tools for Recruiting Healthcare Providers

To understand influences on rHCP behavior, surveys and interviews were developed based on the Theoretical Domains Framework and Capability, Opportunity, and Motivation Behaviour Framework [44]. Surveys were developed to be administered at the following timepoints during the program: prior to rHCP education (HCP survey 2), after the rHCP had been recruiting for 8–12 weeks (HCP survey 3), and toward the end of the study (HCP survey 4). A subset of purposely sampled rHCPs, including higher and lower recruiters, and rHCPs from different geographic areas, were invited to take part in an interview. Table 2 provides further detail of these rHCP data collection tools and the focus for each. 

##### Data Collection Tools for People Offered Screening

**Figure 3:**
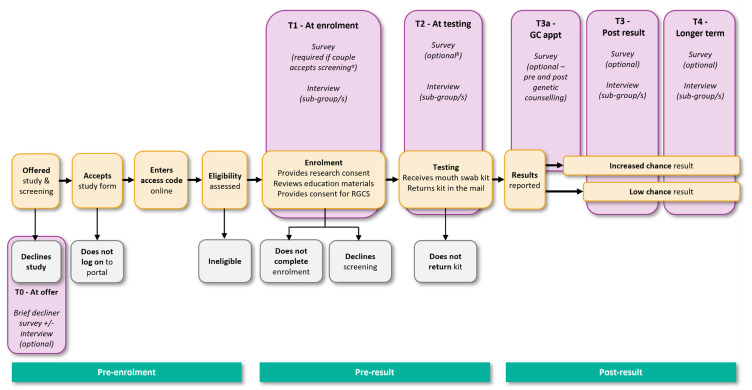
Data collection timepoints for people offered reproductive genetic carrier screening in the Mackenzie’s Mission study.

The first step in developing the participant data collection tools involved identifying the key study time points (see Figure 3): at invitation to take part in MM (T0); at enrollment (T1); at testing, i.e., around the time when the reproductive couple provides samples for RGCS (T2); at the time of the genetic counseling appointment (for increased chance couples, T3a); 3 months post result (T3); and 12 months post result (T4). Surveys and interview guides were developed based on previous research in this area, and the research and clinical experience of the study team. The surveys include a combination of validated scales and purpose-designed questions. Table 3 summarizes the data collection tools developed for each of the timepoints. 

^a^ Participants who do not complete the enrollment or decline screening may also complete this survey.

^b^ Participants who do not return their mouth swab kit may also complete this survey.

**Table 3:** Data collection for people offered reproductive genetic carrier screening in the Mackenzie’s Mission study.

Stage of Participation	Time Point	Data Collection Method	Focus Area	Detail Including Standard Measure Scales
Pre-enrollment	T0 At offer	Survey	Reason(s) for declining the offering of MM Decision-making	Brief demographics Reason(s) for declining Interest in participating in an interview
		Interview		Experience of being invited to take part in Mackenzie’s Mission Decision-making process and factors influencing the decision to decline Attitudes toward RGCS
Pre result	T1 At enrollment	Survey	Participant demographics Knowledge of RGCS Response to being offered RGCS Decision-making Motivations to accept or decline RGCS	Detailed demographics Knowledge check pre and post the education module ^a^ Decision aid ^b^ [42] Factors influencing the decision to accept or decline RGCS State Trait Anxiety Inventory (STAI)—trait anxiety (day-to-day anxiety) measured using the 20-item T-anxiety scale from the STAI Form Y [45] ^c^ and state anxiety (anxiety about a current event) measured using the 6-item STAI-6 [46] ^d^ Australian Quality of Life Instrument (AQoL-4D) ^e^ [47]
		Interview		Experience of being invited to take part in MM Decision-making process and factors influencing decision Broader attitudes toward RGCS
	T2 At testing	Survey	Decision-making at the time of providing the genetic sample	Motivations and decision-making process and approach Decisional Conflict Scale ^f^ [48] STAI-6
Post result	T3a At genetic counseling	Survey	Genetic counseling outcomes (increased chance couples only) pre- and post-appointment	Genomics Outcome Scale ^g^ [49,50] STAI-6
	T3 3 months post result	Survey	Understanding and making meaning of the result	Multidimensional Measure of Informed Choice (MMIC) ^h^ (repetition of portal knowledge questions and attitudes sub-scale developed by Marteau, et al. [51], and adapted by Martyn, et al. [52] Decision Regret Scale ^i^ [53] Professional support Reproductive plans Genomics Outcome Scale STAI-6 AQoL-4D Broader attitudes toward RGCS
		Interview		Use of the information in reproductive decision-making and family communication of genetic information
	T4 12 months post result	Survey	Long-term psychosocial impact of results and reproductive intentions/outcomes	Knowledge retention (repetition of portal knowledge questions) Decision Regret Scale Reproductive intentions and outcomes Professional and social support Family communication STAI-6 AQoL-4D Broader attitudes toward RGCS
		Interview		Use of the information in reproductive decision-making and family communication of genetic information

^a^ Knowledge: 10 purpose-designed statements about RGCS, participants respond with “True”, “False” or “Unsure”. ^b^ Decision aid: Scored on a continuous scale (15–75), where ≤44 = leaning away from screening, 45 = unsure and ≥46 leaning toward screening [42]. ^c^ Participants respond to statements about how they generally feel using a Likert scale in which 1 = almost never and 4 = almost always. Scores are summed to produce a total score ranging from 20–80 [45]. ^d^ Participants respond as according to how they feel in the present moment. The mean score is multiplied by 20 to produce a total score ranging from 20–80 [46]. Higher scores indicate greater anxiety. ^e^ Measures quality of life across four domains (3 items in each): independent living, relationships, mental health and senses. A combined utility score is derived, where 1 = best health state and −0.04 = worst health state [47]. ^f^ Consists of 16 items across 5 subscales (informed, values clarity, support, uncertainty and effective decision). Participants respond to statements using a Likert scale in which 0 = strongly agree and 4 = strongly disagree. Total scores are converted to a range from 0 to 100, where <25 = no decisional conflict, 25–37.5 = moderate decisional conflict and >37.5 = high decisional conflict [48]. ^g^ Measures empowerment as an outcome of genetic counseling. Participants respond to 6 statements using a Likert scale in which 1 = strongly disagree and 5 = strongly agree. Scores range from 0–5 with higher scores = greater empowerment [49]. ^h^ The attitudes dimension was administered within the decision aid and at T3. ^i^ Contains 5 statements with 5 response options (1 = strongly agree and 5 = strongly disagree). Scores range from 0–100 with higher scores indicating greater regret [53].

##### Data Collection Tools for the Study Team

Data collection tools were developed to collect key implementation data from the MM study team. These included: the generation of a standing agenda item in most study team meetings that asked for implementation insights; a guide for interviews with members of the study team that explored barriers and enablers to the implementation of the RGCS program, and lessons learnt; and a time capsule survey that recorded the study team’s predictions of the research objectives and assessed whether these were achieved at the end of the study. From the implementation science perspective, there was a specific focus on collecting the experiential knowledge of the genetic counselors who were recruiting and supporting rHCPs, via regular meetings with the study genetic counselors. 

#### 2.5.6. Designing the Reproductive Genetic Carrier Screen and Establishing Screening Processes

A key element in the pre-implementation phase was designing the MM RGCS and establishing the screening process. A simultaneous screening approach was chosen to enable the screening of both reproductive partners at the same time, with a report providing a combined assessment of reproductive risk for the reproductive couple. Using this approach, individual genetic carrier status for autosomal recessive conditions is not reported [54,55]. This is based on the observation that, when screening for a large number of genes, most people are genetic carriers for at least one condition [56], but the majority (around 98%) of reproductive couples are not genetic carriers for the same condition(s); thus, together, they have a low chance of having children with the conditions screened. With a large gene list, the individual analysis and reporting of individual genetic carrier results is burdensome for laboratories and would create a heavy demand on genetic counseling services. For example, one study showed the median time to provide genetic counseling to an individual following expanded RGCS is 64 min [57]. Limiting the reporting of variants to couples identified to have an increased chance of having an affected child allows the focus of resources to be directed toward those couples who can benefit the most from the screening. For X-linked conditions, an analysis is performed for the female member of the couple, as this result allows for the assessment of the chance of an X-linked condition affecting their children. There are very rare, X-linked conditions for which males typically have mild to no clinical features, whereas females can be severely affected; for example *EFNB1*, which is associated with craniofrontonasal dysplasia and is included in the MM gene list. There are also some genes located in the pseudoautosomal regions of the X and Y chromosomes, for example *SHOX*; biallelic pathogenic variants are associated with Langer mesomelic dysplasia and the gene is included in the MM gene list. These regions are the same in both the X and Y chromosomes and, from an inheritance perspective, behave as though they are on one of the autosomes (chromosomes 1–22), making it necessary to analyze the data from both partners, as for autosomal recessive conditions. 

The screening assays were developed based on published methods with a focus on providing clinically useful information while minimizing the potential harms and risks. Laboratory testing was provided by three National Association of Testing Authorities (NATA, [58]) clinically accredited laboratories: NSW Health Pathology, Randwick Genomics Laboratory in NSW; Victorian Clinical Genetics Services (VCGS) in VIC; and PathWest Laboratory Medicine in WA. A collaborative partnership between the three laboratories was established to enable the sharing of experiences and expertise through the development and implementation of MM. 

The process for selecting the genes to include in the MM RGCS is described elsewhere [59]. Briefly, the goal was to create a list best suited to the Australian healthcare system, the population screening model of MM and Australian societal values. This process was overseen by the Gene Selection Committee and included genes that are associated with severe childhood-onset conditions. When testing commenced, the initial gene list comprised approximately 1300 genes associated with around 750 genetic conditions. A process for regular review was established to allow for genes to be added and removed from the list during the study; the final version of the list used during the study includes 1281 genes (Supplementary File S3). Several important factors were considered when developing the gene list, including that many genes are associated with conditions of varying severity. The identification of couples with an increased chance of having children with a mild condition, with a minimal impact on quality of life, was not the intention of the study. Genetic variants known to be associated with a mild form of a condition are not reported. An example is *CFTR* p.Arg117His (c.350G > A ), which is not considered reportable unless the *CFTR* c.1210-12T [5_9](‘IVS8 polyT’) 5T variant is also identified in the individual [60].

In Australia, approximately 25 autosomal recessive genetic conditions are included in newborn screening. Panels differ between states and territories, although the Australian Federal Government has announced plans to unify and expand the newborn screening program. Several genes associated with conditions that have an effective treatment were included on the MM gene list, including some conditions screened as part of newborn screening. This is because, for some conditions, there may be benefit in initiating treatment prior to newborn screening results being returned (e.g., *IVD*, which is associated with isovaleric acidemia) or for which the treatment is associated with significant burdens [59]. Genes associated with conditions currently included in newborn screening for which the treatment is effective and considered less burdensome (e.g., *TSHR*, which is associated with congenital hypothyroidism) were excluded from the MM gene list [59].

Additionally, one gene (*BRCA2*) was excluded from the MM gene list despite its association with Fanconi anemia, complementation group D1 [59]. Heterozygous *BRCA2* pathogenic variants are associated with an increased chance of developing cancers such as breast and ovarian cancer, the implications of which were considered outside the scope of the study [59]. Research is being conducted to explore participant attitudes toward receiving personal health information from RGCS.

To minimize barriers, screening was designed to be performed on mouth swab samples, which could be posted directly to the reproductive couple and returned by mail. The mouth swab sample kits include user-friendly instructions and a link to a video demonstrating how to self-collect the sample. This approach is a convenient, pain-free sampling method, enabling couples to consider RGCS and provide samples in their own time. This approach also ensured that paired samples are received at the same time, reducing the requirement in the laboratory for storing and matching samples at a later date, as well as having to contact either member of the couple to remind them to send in their samples. Additionally, the quantity and quality of DNA obtained from the swabs is confirmed to be sufficient for all required tests, with a low rate of failure and an infrequent need for recollections. A process was established to assess family and personal history information provided by participants, to ensure the RGCS is appropriate for those couples for whom there is a family or personal history of a condition associated with a gene on the MM gene list. A section was included in the electronic study database to enable the collection of information about the RGCS results and details on variant curation.

### 2.6. Phase 2—Implementation

As described in Figure 2, Phase 2 consists of two stages. During Phase 2a (2020), a small recruitment pilot was first undertaken in order to trial and refine the online enrollment and RGCS processes. The RGCS program was then implemented in four states/territories; this enabled the various aspects of the RGCS program to be further refined to ensure seamless implementation in Phase 2b (2021–2022), where the RGCS program was extended to all other Australian states and territories.

#### 2.6.1. Healthcare Provider Recruitment

Contact was made with HCPs and information about the study was provided either in person or via letter, telephone, fax and/or email. For interested HCPs, a link to the HCP survey 2 was sent via email and education was provided through either: a presentation by a study genetic counselor in person or via video conference, a recorded presentation, or an online education module. Some of these rHCPs participated in a trial of education approaches undertaken by the study implementation science team which will be described and reported separately. rHCPs were provided with their own batch of hard copy and/or electronic study invitation/test request forms, each with a unique access code for the participant portal linked to them. Once the rHCP had completed the education module and received their forms, they were able to start recruiting participants.

Recruitment rates for each rHCP have been closely monitored for the purpose of evaluating the geographic reach of the study and to ensure the representativeness of the sample. Where targets have been achieved for geographic regions, recruitment has ceased in that region or rHCPs have been restricted to recruiting patients from certain postcodes. Regular email newsletters/bulletins are sent to rHCPs summarizing study progress and key updates.

During Phase 2, the implementation science research team and Operations team co-designed and implemented several strategies to support rHCPs to offer RGCS, including a video demonstrating how to offer RGCS, posters for waiting and clinic rooms, regular contact with the MM study team, and an evaluation of different education strategies. Data from the HCP survey 2 and study genetic counselor experiences were then synthesized to generate modified implementation strategies that were later trialed (to be reported separately).

#### 2.6.2. Inviting People to Take Part in Mackenzie’s Mission

After completing the study’s HCP education module, rHCPs began inviting couples who met the eligibility criteria to take part in the study. The rHCP makes this offer to one or both members of the couple—whoever attends the healthcare appointment. Potential participants are informed the study is investigating people’s views and perspectives about RGCS and that participation involves an offer of free screening. If the offer to take part is accepted, they are given a study invitation/test request form and are asked to follow the instructions on the form to enroll in the study online. The rHCP emphasizes that the purpose of the study is to understand the perspectives of those offered RGCS and that those who do not want to undergo RGCS, or are unsure, are welcome to participate and share their views. Those who decline the invitation to take part are provided with a short, optional decliner survey, including the option to leave their contact details to be invited for an interview, which they are asked to complete and return at the clinic reception. Those who do not complete this survey decline all involvement in the study. Where an rHCP identifies that a participant would benefit from enrolling via a modified enrollment pathway, or where a potential participant requests support to enroll, contact is made with the local study team who facilitate enrollment.

#### 2.6.3. Enrollment

For those wanting to participate in the study, one member of the couple accesses the online participant portal via a web link and enters the unique access code provided on the study invitation form. They are then asked to complete questions to assess their eligibility for the study. If the couple does not meet the eligibility criteria, the portal indicates they are not eligible to take part in the study and are unable to proceed further. If the couple meets the eligibility criteria, the participant is prompted to create an account in the portal. They are then asked to provide the contact details of their reproductive partner, who is automatically emailed a link to create their own account in the participant portal. Both reproductive partners complete the full enrollment process in parallel in their own time; this can be completed in one sitting, or participants can log back into the portal to complete their enrollment later. All participants, regardless of whether they choose to have a screening, complete a set of questions for the program evaluation (T1, Table 3). For a couple to access RGCS through the study, the full enrollment process must be completed by both reproductive partners. A small number of purposively sampled participants who commenced enrollment in the study have been invited to take part in an interview (Table 3).

As shown in Figure 3, there are several points in the study at which participants may drop out of or be lost to follow-up during the enrollment and/or RGCS process. Follow-up is undertaken via email reminders and, if necessary, a phone call from a study genetic counselor. If contact is made and reasons for the incomplete enrollment are noted, participants are recorded as having chosen not to complete enrollment. If enrollment is completed but the RGCS mouth swab kit is not returned, the couple is recorded as having chosen not to complete RGCS. Couples who provide research consent but do not complete the enrollment or RGCS process are still able to be invited to participate in MM research activities (e.g., interviews) unless they request no further contact.

#### 2.6.4. Laboratory Testing

**Figure 4:**
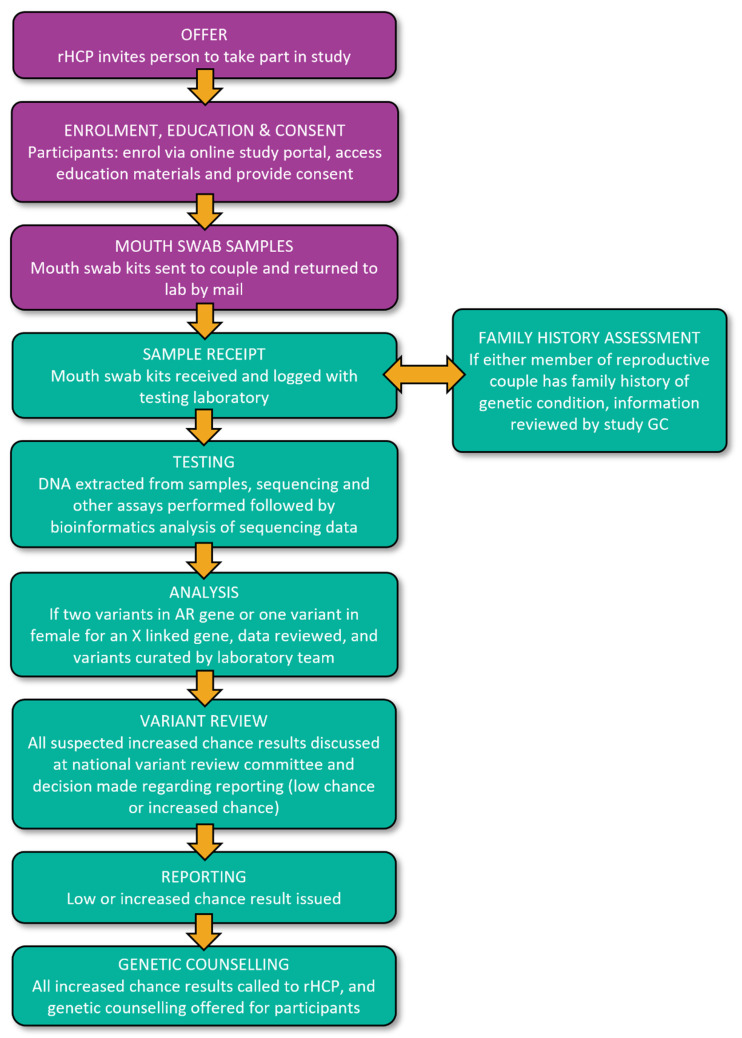
Laboratory testing and management of results in the Mackenzie’s Mission study.

Mouth swab kits are sent by mail to reproductive couples who accept RGCS. Participants return the samples by mail along with the test request form provided by their rHCP. At this time point, they are invited to complete an optional survey (T2, Table 3). Once the samples are received by the laboratory, RGCS is initiated (Figure 4).

All three laboratories use massively parallel sequencing (MPS) assays to sequence all genes in the MM gene list. Separate, additional assays are used for *FMR1* triplet repeat analysis and *SMN1* exon 7 copy number analysis. Two laboratories (NSW Health Pathology and VCGS) use exome sequencing to provide RGCS for participants in ACT, NSW, NT, TAS and VIC, while the third laboratory (PathWest) uses a targeted gene panel to provide RGCS for participants in QLD, SA and WA. The analysis of exome data is bioinformatically restricted to the genes selected for the study. Copy number variation (CNV) analysis is being assessed for some genes; see the Supplementary File S4.

For women with small *FMR1* expansions in the range of 55 to 69 CGG repeats, AGG interruption analysis is performed as this can assist in clarifying reproductive risk in carriers of small premutations [61,62]. Women with 55–64 *CGG* repeats and one or more AGG interruptions, and women with 65–69 repeats and two or more AGG interruptions, are considered to have a low chance of having children with an *FMR1* full mutation [62,63]. For *SMN1* carrier testing, one partner is tested initially; if they have two or more copies of *SMN1*, the other partner is not tested. If the tested partner is found to be a carrier (i.e., has only one copy of *SMN1*), the second partner is tested to determine if the reproductive couple has an increased chance of having children with spinal muscular atrophy.

Sequencing data are assembled, mapped and filtered using each laboratory’s bioinformatics pipeline. Standard filtering criteria are used by all three laboratories, considering data including the predicted variant impact and variant frequency in population databases. The curation of all filtered variants follows the American College of Medical Genetics (ACMG) guidelines, appropriately modified to take into account the specific setting of testing for carrier status [64]. An increased chance result is issued if pathogenic or likely pathogenic variants (ACMG Class 5 and 4) that meet the criteria for reporting in the study are identified in the same autosomal gene in both members of a reproductive couple, and/or in a gene on the X chromosome in the female partner. In RGCS, most reproductive couples have no family history of the genetic condition and no clinical features relating to carrier status. Therefore, considerable caution is taken regarding the reporting of variants. In line with the usual clinical practice for exome sequencing and targeted gene panels, if there is any doubt about the classification of variants, they are reviewed at a weekly national MM Variant Review Committee teleconference meeting, attended by clinical and laboratory staff, at which a consensus decision is made regarding the suitability of the variant for reporting. The weekly Variant Review Committee meeting also serves as the forum for recording all variants that are reported, including those for which a straightforward pathogenic classification is possible. If additional information becomes available after a variant is reported, which may change its classification (e.g., family segregation data), this is also discussed at the Variant Review Committee. Laboratory reports describe the limitations of the assay, including the possibility that variants may not be identified due to areas of poor sequencing or other technical limitations, and that variants may not be able to be classified as likely pathogenic or pathogenic due to the limitations of knowledge at the time of the report. The RGCS consent process includes information about the possibility that a pathogenic variant may not be detected or reported, and low risk results are accompanied by an information sheet that explains these limitations.

#### 2.6.5. Management of Results

Results are reported by the laboratory that performed the testing using a standardized study report template. The report for the reproductive couple conveys either that the couple has a low chance of having children with conditions associated with any of the genes screened, or the couple has an increased chance of having children with one or more conditions. The results of carrier status for each individual within the reproductive couple are not issued and only the combined result for the reproductive couple.

Results indicating a low chance of conditions associated with any of the genes screened are issued to the rHCP by fax, secure email or mail. Both reproductive partners are notified via email that the result has been uploaded to the secure online participant portal. They are then able to use their individual login details to access their report and a factsheet explaining their low chance result. Participants have the option of discussing their result with a study genetic counselor. For those who enrolled via a modified enrollment pathway, the result is provided by a study genetic counselor (with an interpreter as required). Where necessary, correspondence is translated into the relevant language.

When the results indicate an increased chance for one or more of the conditions screened, a study genetic counselor contacts the rHCP to discuss the result. The couple is then contacted directly by the genetic counselor. The genetic counselor discusses the result with the couple by telephone and offers a genetic counseling consultation as soon as practicable, either face-to-face or via telehealth. When participants using one or more donor(s) receive an increased chance result, genetic counseling is available to all involved parties, including those who are not the genetic parents of the current or planned pregnancy but who would be parenting the child. The genetic counseling consultation (provided by a genetic counselor, with a clinical geneticist if required) includes a discussion of the clinical features of the condition, the implications of the result, reproductive options and implications of the information for relatives, including current children, if relevant. The study genetic counselors assist people in adjusting to their knowledge of the results and support their decision-making regarding their reproductive options. If appropriate, access to subspecialists with expertise in the condition is also available (e.g., a pediatric respiratory physician for cystic fibrosis, a pediatric neurologist for spinal muscular atrophy, etc.) and, as available, to the support organization for the relevant condition. A copy of the result is provided to the couple either before, during or after their genetic counseling consultation via email/mail/in person, in line with standard clinical practice.

A range of reproductive options is discussed with and offered to participants who receive an increased chance result, including: PND by chorionic villus sampling or amniocentesis (with the option of pregnancy termination if affected), IVF with PGT-M, using donor gametes/embryos (with RGCS for the donors), adoption/fostering, choosing not to have children/more children or choosing not to have any testing. For MM increased chance couples, PND and PGT-M are available at no charge to the couple so that the study can assess reproductive choices when cost is not a barrier. Funded PND is provided through the Australian public healthcare system and one cycle of IVF with PGT-M is funded through the study in partnership with local IVF services. For all increased chance couples, the local study clinical team provide comprehensive clinical support around the consideration of each of these options as well as psychosocial support, referral to specialists if there are considerations regarding pregnancy management, referral for the termination of pregnancy if requested, or testing, clinical assessment and management of the baby after birth.

Increased chance couples are asked to complete a survey before and after genetic counseling (T3a, Table 3). Approximately three months post result, all participants are invited to complete an online survey, and again approximately 12 months post result (T3 and T4, respectively, Table 3). Subsets of participants are also invited to take part in a semi-structured interview at either of these timepoints.

### 2.7. Phase 3—Post-Implementation: Assessing Outcomes

Phase 3 will commence in the second half of 2022 and will involve an analysis of the quantitative and qualitative data, and ultimately will report the study findings to the Australian Government. The results are informing an application for publicly funded expanded RGCS in Australia which has been submitted through the Medical Services Advisory Committee (MSAC) [65], an entity that advises the Australian Minister for Health on tests that should be funded through Medicare, the publicly funded universal healthcare insurance scheme in Australia.

#### 2.7.1. Proposed Analysis

This observational study involves a mixed-methods approach, integrating data from across the psychosocial and epidemiology, implementation science, and health economics research streams, to assess the acceptability and feasibility of the developed RGCS program. An effort has been made to capture a wide variety of attitudes and views, including of those who decline the offer of RGCS. These findings will be presented against the background of the bioethics analysis, which has been undertaken throughout the project.

##### Quantitative Analysis

Quantitative data will be analyzed using the statistical software packages, STATA 16 and SPSS. Descriptive statistics will be used to summarize the program outcomes including the RGCS uptake, frequency of increased chance couples and reproductive decisions made by these couples. Detailed participant characteristics will be reported, with the representativeness of the participant sample compared to Australian population data assessed using a minimum dataset (e.g., socioeconomic status via the Index of Relative Advantage and Disadvantage quintile).

Data will be summarized using descriptive statistics (e.g., means, standard deviations, medians, interquartile ranges, frequency distributions and 95% confidence intervals). Inferential analysis will include univariable analysis such as χ^2^ and Fisher exact tests, unadjusted logistic regressions for categorical outcomes, and independent, two-tailed *t*-tests or non-parametric Mann–Whitney U tests for continuous outcomes. A comparison between participants who had RGCS and participants who did not proceed with RGCS after initiating enrollment will be performed using multivariable logistic regression, adjusting for significant covariates (*p* < 0.1 in the univariable analysis).

##### Psychosocial Outcomes

The primary psychosocial outcome is anxiety, as measured by the State Trait Anxiety Inventory (STAI) [45]. The STAI is administered from T1–T4 (see Table 3) to enable analysis within and across time points. Other psychosocial outcomes to be investigated include RGCS knowledge, decisional conflict and decision regret. *A priori* confounders (e.g., the highest level of education attained) and covariates associated with the outcome at *p* < 0.1 in univariable analyses will be included in multivariable analyses. These will take the form of logistic regression modeling for binary outcomes. As these will be planned analyses, probability values of <0.05 will be considered statistically significant. To account for missing data, response bias will be assessed using between-group comparisons of responders and non-responders at different time points. Inverse probability weights from propensity scores will then be generated.

##### Implementation Science

The implementation science research team is using implementation science and behavior change theories, e.g., the Consolidated Framework for Implementation Research [66] and Theoretical Domains Framework [67], to understand the effectiveness of the implemented RGCS program. Barriers were identified earlier in the implementation phase of the study and implementation interventions were developed to overcome these (to be reported separately). A subset of these strategies has been evaluated, with input from the study genetic counselors, for acceptability and feasibility. Enablers that contributed to the successful implementation and effectiveness of rHCP education are also being investigated.

##### Health Economic Analysis

A cost-effectiveness analysis will be conducted taking the costs to the healthcare system as the perspective. A decision tree model with probabilities based on outcomes from MM, costs from the Medicare Benefits Schedule and Pharmaceutical Benefits Schedule, and published data on lifetime costs of disease will be developed. Costs to the health system include the costs associated with IVF decisions, termination of pregnancy, and the expected future healthcare costs of maintaining a live birth with a severe inherited genetic condition. All costs will be standardized to 2022 values. The AQoL-4D will be scored using the Australian values [47] for both parents to identify any changes in quality of life over the 12 months of follow-up. A hypothetical comparator will be used to estimate costs and outcomes for the counterfactual of ‘usual care in the absence of the MM program’. This counterfactual will assign quality of life weights to both parents using population norms [68]; for those parents who have a live birth of a child with a genetic condition, a lower quality of life weight identified from MM and the literature will be assigned for the duration of the expected survival of the child. A quality of life weight for the child with a genetic condition will also be assigned for their expected survival. Quality of life and survival will be combined into quality-adjusted life years (QALYs). All future expected costs and QALYs will be converted into today’s values, discounted using a 5% rate based on Australian policy requirements, as stipulated by MSAC [65]. The difference in QALYs and costs for the MM participants and the counterfactual will be calculated and reported as an incremental cost-effectiveness ratio. TreeAge Pro (2018) will be used in this analysis.

##### Qualitative Analysis

Interviews will be transcribed with participants assigned a pseudonym or participant code. Data will be co-coded and managed using NVivo (QSR International). Thematic analysis, or content analysis as appropriate, will be used for the analysis of qualitative interview data. Data gathered from rHCPs and the MM study team will be analyzed using appropriate implementation science and behavior change frameworks.

#### 2.7.2. Use and Analysis of Genomic Data

When providing consent to RGCS as part of the study, participants are also asked to provide consent to share their de-identified genomic data to advance the scientific knowledge of genetic conditions. Genomic data generated by the study are stored by the three laboratories as required by the Australian regulatory body, the National Pathology Accreditation Advisory Council (NPAAC). Additionally, the data are stored in a re-identifiable format, in a secure central data repository managed by Australian Genomics. Through a data access request process, de-identified genomic data can be shared with other ethically approved research studies and with genomic databases, in accordance with a participant’s data sharing preferences. The project is currently working with collaborators to generate harmonized summary data regarding allele frequencies which can be made openly available to researchers and diagnostic laboratories. Summarized data regarding allele frequencies will also be made available to researchers and diagnostic laboratories via the Centre for Population Genomics.

## 3. Discussion

This study is the first of its kind to investigate the acceptability and feasibility of a government-funded RGCS program screening over 1000 genes. The rationale behind the Australian Federal Government’s funding of the MM research study is to investigate how best to deliver a potential, future, publicly funded, easily accessible RGCS program. A strength of the study design is the ability to capture attitudes and experiences across various time points, including at the offer of screening and post result. The study will provide important data to inform decisions about the implementation of an ethically robust national RGCS program and inform the development of models for delivering carrier screening services. Findings will also be presented in scientific publications, at national and international conferences, disseminated to participants, and a summary will be available on the study website [23]. To support the translation of study findings into health services policy and clinical practice, an application has been prepared and submitted to the MSAC. The aim of the application is to request government funding of RGCS for a widely accessible and comprehensive screening program. In addition, the study findings will inform the preparation and submission of recommendations regarding the implementation of a population-wide RGCS program to both Federal Australian and State/Territory Governments, and professional bodies including RANZCOG, RACGP, RCPA and HGSA.

## Figures and Tables

**Table 1:** Inclusion and exclusion criteria.

Inclusion Criteria	Exclusion Criteria
Planning to become pregnant or in early pregnancy (9 weeks and 6 days of gestation or less ^a^)	Pregnant and 10 weeks of gestation or greater ^a^
Both members of the reproductive couple are available to participate in the study and provide a sample for testing	Only one member of the reproductive couple agrees to participate in the study Both members of the reproductive couple are not available to be tested at the same time
If a donor(s) is being used for the pregnancy, the donor(s) must be available to provide a sample for testing and consent to having RGCS	A donor(s) is being used for the pregnancy and the donor(s) is/are not available for testing, or an anonymous donor is being used A donor(s) is being used for the pregnancy and not all people involved in the pregnancy agree to participate in the study
Both members of the reproductive couple are aged 18 years or over	One or both members of the reproductive couple are less than 18 years old

^a^ To ensure results are returned in an appropriate timeframe, gestation cut-offs for each state/territory were established for cases where screening had occurred in early pregnancy. The gestation cut-off for enrollment in MM and undergoing RGCS has changed throughout the course of the study. When participant recruitment first commenced, only non-pregnant couples were eligible to enroll in the study. As the study progressed, recruitment numbers and test turnaround times at the three laboratories providing the RGCS were carefully monitored. The recruitment of couples in early pregnancy began when test turnaround times had become sufficiently fast enough to be able to return results in an ethically and clinical acceptable timeframe. Impacts of the SARS-CoV-2 pandemic, workplace restrictions and postage considerations also required reassessment of gestation cut-offs at various points during the study. For simplicity, we refer to a gestation cut-off of less than 10 weeks at enrollment and less than 11 weeks at sample receipt by the laboratory, as these have been the latest possible gestation cut-offs utilized and evaluated during the study.

**Table 2:** Data collection tools for recruiting HCPs in the Mackenzie’s Mission study ^a^.

Timepoint	Data Collection Tool(s)	Main Focus of Data Collection
Prior to education to become an rHCP	HCP survey 2	Potential barriers to implementing RGCS Factors that might influence confidence/ability to refer people for RGCS Readiness to change practice Knowledge of concepts relating to RGCS
Eight to 12 weeks after rHCP education	HCP survey 3 HCP interview guide ^b^	Knowledge of concepts relating to RGCS and study processes Attitudes toward RGCS and factors influencing recruitment, such as knowledge, ability and confidence Views on factors that may influence patient accessibility to RGCS, including socioeconomic status, geographic location, language, cultural background, health literacy and disability Reasons for offering/not offering the study to patients and the reasons patients provide when they decline to take part
Toward the end of the study	HCP survey 4	Rating identified facilitators to offering RGCS

^a^ HCP survey 1 is not included in this table as it was administered to HCPs prior to their involvement in MM. ^b^ Interviews are being conducted by telephone or video conference, and audio-recorded.

## Data Availability

Study data collection tools, education modules and consent content are available from the corresponding author upon reasonable request.

## Supplementary Materials

The following supporting information can be downloaded at: https://www.mdpi.com/article/10.3390/jpm12111781/s1, File S1: List of HREC approvals; File S2: Study invitation form; File S3: MM gene and condition list_V2.2_23.07.2021; File S4: Laboratory methods [69,70,71,72,73].

## References

[1] Delatycki M.B., Laing N., Moore S., Emery J., Archibald A.D., Massie J., Kirk E. (2019). Preconception and antenatal carrier screening for genetic conditions: ‘The critical role of general practitioners’. Aust. J. Gen. Pract..

[2] Delatycki M.B., Alkuraya F., Archibald A.D., Castellani C., Cornel M., Grody W.W., Henneman L., Ioannides A.S., Kirk E., Laing N. (2020). International perspectives on the implementation of reproductive carrier screening. Prenat. Diagn..

[3] Beauchamp K.A., Johansen Taber K.A., Muzzey D. (2019). Clinical impact and cost-effectiveness of a 176-condition expanded carrier screen. Genet. Med..

[4] Ropers H.H. (2012). On the future of genetic risk assessment. J. Community Genet..

[5] Dive L., Newson A.J. (2021). Ethics of reproductive genetic carrier screening: From the clinic to the population. Public Health Ethics.

[6] McClaren B.J., Metcalfe S.A., Amor D.J., Aitken M., Massie J. (2011). A case for cystic fibrosis carrier testing in the general population. Med. J. Aust..

[7] Kraft S.A., Duenas D., Wilfond B.S., Goddard K.A.B. (2019). The evolving landscape of expanded carrier screening: Challenges and opportunities. Genet. Med..

[8] Westemeyer M., Saucier J., Wallace J., Prins S.A., Shetty A., Malhotra M., Demko Z.P., Eng C.M., Weckstein L., Boostanfar R. (2020). Clinical experience with carrier screening in a general population: Support for a comprehensive pan-ethnic approach. Genet. Med..

[9] Henneman L., Borry P., Chokoshvili D., Cornel M.C., van El C.G., Forzano F., Hall A., Howard H.C., Janssens S., Kayserili H. (2016). Responsible implementation of expanded carrier screening. Eur. J. Hum. Genet..

[10] American College of Obstetricians and Gynecologists (2017). Committee Opinion No. 690 Summary: Carrier Screening in the Age of Genomic Medicine. Obstet. Gynecol..

[11] (2019). The Royal Australian and New Zealand College of Obstetricians and Gynaecologists. Genetic Carrier Screening.

[12] Beard C., Amor D., Di Pietro L., Archibald A.D. (2016). “I’m Healthy, It’s Not Going To Be Me”: Exploring experiences of carriers identified through a population reproductive genetic carrier screening panel in Australia. Am. J. Med. Genet. A.

[13] Plantinga M., Birnie E., Abbott K., Sinke R., Lucassen A., Schuurmans J., Kaplan S., Verkerk M., Ranchor A., van Langen I. (2016). Population-based preconception carrier screening: How potential users from the general population view a test for 50 serious diseases. Eur. J. Hum. Genet. EJHG.

[14] Schuurmans J., Birnie E., Ranchor A.V., Abbott K.M., Fenwick A., Lucassen A., Berger M.Y., Verkerk M., van Langen I.M., Plantinga M. (2020). GP-provided couple-based expanded preconception carrier screening in the Dutch general population: Who accepts the test-offer and why?. Eur. J. Hum. Genet..

[15] Ong R., Howting D., Rea A., Christian H., Charman P., Molster C., Ravenscroft G., Laing N.G. (2018). Measuring the impact of genetic knowledge on intentions and attitudes of the community towards expanded preconception carrier screening. J. Med. Genet..

[16] Ioannou L., Delatycki M., Massie J., Hodgson J., Lewis S. (2015). “Suddenly Having two Positive People who are Carriers is a Whole New Thing”—Experiences of Couples Both Identified as Carriers of Cystic Fibrosis Through a Population-Based Carrier Screening Program in Australia. J. Genet. Couns..

[17] Tardif J., Pratte A., Laberge A.M. (2018). Experience of carrier couples identified through a population-based carrier screening pilot program for four founder autosomal recessive diseases in Saguenay-Lac-Saint-Jean. Prenat. Diagn..

[18] Archibald A.D., Hickerton C., Jaques A., Wake S., Cohen J., Metcalfe S. (2013). “It’s about having the choice”: Stakeholder perceptions of population-based genetic carrier screening for fragile X syndrome. Am. J. Med. Genet. A.

[19] Metcalfe S., Martyn M., Ames A., Anderson V., Archibald A., Carter R., Cohen J., Cotter M., Dang W., Delatycki M. (2017). Informed decision making and psychosocial outcomes in pregnant and nonpregnant women offered population fragile X carrier screening. Genet. Med..

[20] Archibald A.D., Smith M., Burgess T., Scarff K., Elliott J., Hunt C., McDonald Z., Barns-Jenkins C., Holt C., Sandoval K. (2018). Reproductive genetic carrier screening for cystic fibrosis, fragile X syndrome, and spinal muscular atrophy in Australia: Outcomes of 12,000 tests. Genet. Med..

[21] Leibowitz R., Lewis S., Massie J., Emery J.D., Smith M., Delatycki M.B., Archibald A.D. (2022). Reproductive genetic carrier screening for cystic fibrosis, fragile X syndrome and spinal muscular atrophy: Patterns of community and healthcare provider participation in a Victorian screening program. Aust. J. Prim. Care.

[22] Robson S.J., Caramins M., Saad M., Suthers G. (2020). Socioeconomic status and uptake of reproductive carrier screening in Australia. Aust. N. Z. J. Obstet. Gynaecol..

[23] Mackenzie’s Mission. https://www.mackenziesmission.org.au/.

[24] Casella R. (2020). Mackenzie’s Mission.

[25] Australian Genomics. https://www.australiangenomics.org.au/.

[26] Dive L., Archibald A.D., Newson A.J. (2021). Ethical considerations in gene selection for reproductive carrier screening. Hum. Genet..

[27] Dive L., Newson A.J. (2020). Ethical issues in reproductive genetic carrier screening. Med. J. Aust..

[28] Dive L., Newson A.J. (2021). Reproductive carrier screening: Responding to the eugenics critique. J. Med. Ethics.

[29] Newson A.J., Dive L. (2022). Taking seriousness seriously in genomic health. Eur. J. Hum. Genet..

[30] Newson A.J., Dive L., Cini J., Hurley E., Farrar M.A. (2022). Ethical aspects of the changing landscape for spinal muscular atrophy management in Australia. Aust. J. Gen. Pract..

[31] Best S., Long J., Theodorou T., Hatem S., Lake R., Archibald A.D., Freeman L., Braithwaite J. (2021). Health practitioners’ perceptions of the barriers and enablers to the implementation of reproductive genetic carrier screening: A systematic review. Prenat. Diagn..

[32] Taylor N., Parveen S., Robins V., Slater B., Lawton R. (2013). Development and initial validation of the Influences on Patient Safety Behaviours Questionnaire. Implement. Sci..

[33] Michie S., Johnston M., Abraham C., Lawton R., Parker D., Walker A. (2005). Making psychological theory useful for implementing evidence based practice: A consensus approach. BMJ Qual. Saf..

[34] Harris P.A., Taylor R., Minor B.L., Elliott V., Fernandez M., O’Neal L., McLeod L., Delacqua G., Delacqua F., Kirby J. (2019). The REDCap consortium: Building an international community of software platform partners. J. Biomed. Inform..

[35] Harris P.A., Taylor R., Thielke R., Payne J., Gonzalez N., Conde J.G. (2009). Research electronic data capture (REDCap)—A metadata-driven methodology and workflow process for providing translational research informatics support. J. Biomed. Inform..

[36] Australian Bureau of Statistics Births, Australia, 2016. https://www.abs.gov.au/ausstats/abs@.nsf/Previousproducts/3301.0Main%20Features12016?opendocument&tabname=Summary&prodno=3301.0&issue=2016&num=&view=.

[37] Health A.I.o. (2018). Welfare. Australia’s Mothers and Babies 2016—In Brief.

[38] Archibald A.D., Hickerton C., Wake S., Jaques A., Cohen J., Metcalfe S. (2016). “It gives them more options”: Preferences for preconception genetic carrier screening for fragile X syndrome in primary healthcare. J. Community Genet..

[39] Valente G.M., Amor D.J., Ioannou L.J., Archibald A.D. (2020). Factors influencing medical practitioner participation in population carrier screening for cystic fibrosis. Aust. N. Z. J. Obs. Gynaecol..

[40] Nagle C., Gunn J., Bell R., Lewis S., Meiser B., Metcalfe S., Ukoumunne O.C., Halliday J. (2008). Use of a decision aid for prenatal testing of fetal abnormalities to improve women’s informed decision making: A cluster randomised controlled trial [ISRCTN22532458]. BJOG Int. J. Obstet. Gynaecol..

[41] Nagle C., Lewis S., Meiser B., Metcalfe S., Carlin J.B., Bell R., Gunn J., Halliday J. (2006). Evaluation of a decision aid for prenatal testing of fetal abnormalities: A cluster randomised trial [ISRCTN22532458]. BMC Public Health.

[42] King E., Halliday J., Archibald A.D., Delatycki M., Barlow-Stewart K., Newson A.J., McClaren B.J. (2022). Development and use of the Australian reproductive genetic carrier screening decision aid. Eur. J. Hum. Genet..

[43] Haas M.A., Teare H., Prictor M., Ceregra G., Vidgen M.E., Bunker D., Kaye J., Boughtwood T. (2021). ‘CTRL’: An online, Dynamic Consent and participant engagement platform working towards solving the complexities of consent in genomic research. Eur. J. Hum. Genet..

[44] Michie S., Van Stralen M.M., West R. (2011). The behaviour change wheel: A new method for characterising and designing behaviour change interventions. Implement. Sci..

[45] Spielberger C.D., Gorsuch R.L., Lushene R., Vagg P.R., Jacobs G.A. (1983). Manual for the State-Trait Anxiety Inventory.

[46] Marteau T., Bekker H. (1992). The development of a six-item short-form of the state scale of the Spielberger State—Trait Anxiety Inventory (STAI). Br. J. Clin. Psychol..

[47] Hawthorne G., Richardson J., Osbourne R. (1999). The Assessment of Quality of life (AQoL) instrument: A psychometric measure of Health-Related Quality of Life. Qual. Life Res..

[48] O’Connor A. (1993). User Manual—Decisional Conflict Scale (16 Item Statement Format).

[49] Grant P., Pampaka M., Payne K., Clarke A., McAllister M. (2018). Developing a short-form of the Genetic Counselling Outcome Scale: The Genomics Outcome Scale. Eur. J. Med. Genet..

[50] McAllister M., Wood A., Dunn G., Shiloh S., Todd C. (2011). The Genetic Counseling Outcome Scale: A new patient-reported outcome measure for clinical genetics services. Clin. Genet..

[51] Marteau T.M., Dormandy E., Michie S. (2001). A measure of informed choice. Health Expect..

[52] Martyn M., Anderson V., Archibald A., Carter R., Cohen J., Delatycki M., Donath S., Emery J., Halliday J., Hill M. (2013). Offering fragile X syndrome carrier screening: A prospective mixed-methods observational study comparing carrier screening of pregnant and non-pregnant women in the general population. BMJ Open.

[53] Brehaut J.C., O’Connor A.M., Wood T.J., Hack T.F., Siminoff L., Gordon E., Feldman-Stewart D. (2003). Validation of a decision regret scale. Med. Decis. Mak. Int. J. Soc. Med. Decis. Mak..

[54] Schuurmans J., Birnie E., van den Heuvel L.M., Plantinga M., Lucassen A., van der Kolk D.M., Abbott K.M., Ranchor A.V., Diemers A.D., van Langen I.M. (2019). Feasibility of couple-based expanded carrier screening offered by general practitioners. Eur. J. Hum. Genet..

[55] Birnie E., Schuurmans J., Plantinga M., Abbott K.M., Fenwick A., Lucassen A., Berger M.Y., Van Langen I.M., Ranchor A.V. (2021). Couple-based expanded carrier screening provided by general practitioners to couples in the Dutch general population: Psychological outcomes and reproductive intentions. Genet. Med..

[56] Bell C.J., Dinwiddie D.L., Miller N.A., Hateley S.L., Ganusova E.E., Mudge J., Langley R.J., Zhang L., Lee C.C., Schilkey F.D. (2011). Carrier testing for severe childhood recessive diseases by next-generation sequencing. Sci. Transl. Med..

[57] Lynch F.L., Himes P., Gilmore M.J., Morris E.M., Schneider J.L., Kauffman T.L., Shuster E., Reiss J.A., Dickerson J.F., Leo M.C. (2018). Time costs for genetic counseling in preconception carrier screening with genome sequencing. J. Genet. Couns..

[58] NATA. https://nata.com.au/.

[59] Kirk E.P., Ong R., Boggs K., Hardy T., Righetti S., Kamien B., Roscioli T., Amor D.J., Bakshi M., Chung C.W.T. (2021). Gene selection for the Australian Reproductive Genetic Carrier Screening Project (“Mackenzie’s Mission”). Eur. J. Hum. Genet..

[60] Human Genetics Society of Australasia (2013). Population Based Carrier Screening for Cystic Fibrosis.

[61] Yrigollen C., Durbin-Johnson B., Gane L., Nelson D., Hagerman R., Hagerman P., Tassone F. (2012). AGG interruptions within the maternal FMR1 gene reduce the risk of offspring with fragile X syndrome. Genet. Med..

[62] Nolin S.L., Glicksman A., Ersalesi N., Dobkin C., Brown W.T., Cao R.U., Blatt E., Sah S., Latham G.J., Hadd A.G. (2015). Fragile X full mutation expansions are inhibited by one or more AGG interruptions in premutation carriers. Genet. Med..

[63] Nolin S.L., Sah S., Glicksman A., Sherman S.L., Allen E., Berry-Kravis E., Tassone F., Yrigollen C., Cronister A., Jodah M. (2013). Fragile X AGG analysis provides new risk predictions for 45–69 repeat alleles. Am. J. Med. Genet. Part A.

[64] Richards S., Aziz N., Bale S., Bick D., Das S., Gastier-Foster J., Grody W.W., Hegde M., Lyon E., Spector E. (2015). Standards and guidelines for the interpretation of sequence variants: A joint consensus recommendation of the American College of Medical Genetics and Genomics and the Association for Molecular Pathology. Genet. Med..

[65] Medical Services Advisory Committee Guidelines for Preparing Assessments for the MSAC. http://www.msac.gov.au/internet/msac/publishing.nsf/Content/MSAC-Guidelines.

[66] Damschroder L.J., Aron D.C., Keith R.E., Kirsh S.R., Alexander J.A., Lowery J.C. (2009). Fostering implementation of health services research findings into practice: A consolidated framework for advancing implementation science. Implement. Sci..

[67] Cane J., O’Connor D., Michie S. (2012). Validation of the theoretical domains framework for use in behaviour change and implementation research. Implement. Sci..

[68] Hawthorne G., Korn S., Richardson J. (2013). Population norms for the AQoL derived from the 2007 Australian National Survey of Mental Health and Wellbeing. Aust. N. Z. J. Public Health.

[69] Sadedin S.P., Dashnow H., James P.A., Bahlo M., Bauer D.C., Lonie A., Lunke S., Macciocca I., Ross J.P., Siemering K.R. (2015). Cpipe: A shared variant detection pipeline designed for diagnostic settings. Genome Med..

[70] Sadedin S.P., Ellis J.A., Masters S.L., Oshlack A. (2018). Ximmer: A system for improving accuracy and consistency of CNV calling from exome data. Gigascience.

[71] gnomAD. https://gnomad.broadinstitute.org/.

[72] ClinVar. https://www.ncbi.nlm.nih.gov/clinvar/.

[73] Chen L., Hadd A., Sah S., Filipovic-Sadic S., Krosting J., Sekinger E., Pan R., Hagerman P.J., Stenzel T.T., Tassone F. (2010). An information-rich CGG repeat primed PCR that detects the full range of fragile X expanded alleles and minimizes the need for southern blot analysis. J. Mol. Diagn..

