# Supplementary material for: The Australian Reproductive Genetic Carrier Screening Project (Mackenzie’s Mission): Design and Implementation
Source: J Pers Med. 2022 Oct 28;12(11):1781. doi: 10.3390/jpm12111781 (PMC9698511; doi:10.3390/jpm12111781)
Supplement: Supplementary file 1 [file jpm-12-01781-s001.zip › Supplementary File S1 - List of HREC approvals.pdf]

Supplementary File S1 – List of Human Research Ethics Committee approvals

Approving HREC	Study site(s) included in HREC approval	State/ territory	Study site type			
			Participant recruitment	Laboratory testing	Clinical	Research
The Royal Children's Hospital HREC, VIC (*lead reviewing HREC)*	Campbelltown Hospital	NSW	✓			
	Forster Community Health Service	NSW	✓			
	Hunter Genetics	NSW	✓			
	Liverpool Hospital	NSW	✓			
	NSW Health Pathology	NSW		✓		
	Royal Hospital for Women	NSW	✓			
	Royal Prince Alfred Hospital	NSW	✓			
	Sydney Children's Hospital Randwick	NSW	✓		✓	
	Tamworth Community Health Service	NSW	✓			
	Taree Community Health Service	NSW	✓			
	The Children's Hospital at Westmead	NSW	✓		✓	
	Wagga Wagga Base Hospital	NSW	✓			
	Westmead Hospital	NSW	✓			
	Royal Brisbane and Women's Hospital, including Genetic Health Queensland	QLD	✓		✓	
	Repromed Adelaide	SA	✓			
	Women's and Children's Hospital, including South Australian Clinical Genetics Service	SA	✓		✓	
	Murdoch Children's Research Institute	VIC				✓
	Northern Hospital	VIC	✓			
	Victorian Clinical Genetics Services	VIC	✓	✓	✓	

Approving HREC	Study site(s) included in HREC approval	State/ territory	Study site type			
			Participant recruitment	Laboratory testing	Clinical	Research
The Royal Children's Hospital HREC, VIC (*continued)*	King Edward Memorial Hospital, including Genetic Services of Western Australia	WA	✓		✓	
	PathWest Laboratory Medicine	WA		✓		
	Perth Children's Hospital	WA	✓			
	Private healthcare settings[1] in the Australian Capital Territory, New South Wales, Queensland, South Australia, Victoria, and Western Australia	Various	✓			
Aboriginal Health and Medical Research Council HREC, NSW	Armajun Aboriginal Health Service	NSW	✓			
IVF Australia HREC, NSW	IVF Australia	NSW	✓			
Macquarie University HREC, NSW[2]	Macquarie University	NSW				✓
University of New South Wales HREC, NSW[2]	University of New South Wales	NSW				✓
University of Sydney HREC, NSW[2]	University of Sydney	NSW				✓
HREC of the Northern Territory Department of Health and Menzies School of Health Research, NT	Repromed Darwin	NT	✓			
	Royal Darwin Hospital	NT	✓			
	Private healthcare settings[1] in the Northern Territory (Top End)	NT	✓			
Central Australian HREC, NT	Private healthcare settings[1] in the Northern Territory (Central Australia)	NT	✓			
Royal Brisbane and Women's Hospital HREC, QLD	Aboriginal and Torres Strait Islander Community Controlled Health Organisations that are members of the Queensland Aboriginal and Islander Health Council (QAIHC)	QLD	✓			
Griffith University HREC, QLD[2]	Griffith University	QLD				✓

Approving HREC	Study site(s) included in HREC approval	State/ territory	Study site type			
			Participant recruitment	Laboratory testing	Clinical	Research
Tasmania Health and Medical HREC, TAS	Royal Hobart Hospital, including Tasmanian Clinical Genetics Service	TAS	✓		✓	
	Private healthcare settings[1] in Tasmania	TAS	✓			
Melbourne IVF HREC, VIC	Melbourne IVF	VIC	✓			
Mercy Health HREC, VIC	Mercy Hospital for Women	VIC	✓			
Ramsay Health Care WA|SA HREC, WA/SA	Joondalup Health Campus	WA	✓			
University of Western Australia HREC, WA[2]	University of Western Australia	WA				✓

List of abbreviations

HREC	Human Research Ethics Committee
NSW	New South Wales
NT	Northern Territory
QLD	Queensland
SA	South Australia
TAS	Tasmania
VIC	Victoria
WA	Western Australia

[1] Private healthcare settings include general practice, private obstetrics, private midwifery, private ultrasound, and some fertility services.

[2] The HREC recognised the ethical approval provided by the Royal Children's Hospital HREC in lieu of providing their own ethical review and approval.
