# Supplementary material for: The Australian Reproductive Genetic Carrier Screening Project (Mackenzie’s Mission): Design and Implementation
Source: J Pers Med. 2022 Oct 28;12(11):1781. doi: 10.3390/jpm12111781 (PMC9698511; doi:10.3390/jpm12111781)
Supplement: Supplementary file 1 [file jpm-12-01781-s001.zip › Supplementary File S2 - Study invitation form.pdf]

MACKENZIE'S MISSION
REPRODUCTIVE GENETIC CARRIER SCREENING

STUDY INVITATION FORM

You are invited to take part in Mackenzie's Mission

Mackenzie's Mission is a research study funded by the Australian Federal Government. Through Mackenzie's Mission, people planning a pregnancy or early in pregnancy have the option of screening to find out if they and their reproductive partner have an increased chance of having a child with a serious genetic condition.

Genetic carrier screening as part of this study is free and simple. All participants will have access to information and support throughout the study. We want to understand what people think about genetic carrier screening.

We welcome your participation in the study, even if you are unsure about whether you want to have screening.

HOW TO TAKE PART

Enrol in the study at **mmstudy.org.au**

Your unique access code: 1-A11-111

This access code is only for your use as a couple and must not be passed onto anyone else.

WHAT IS INVOLVED?

Taking part in Mackenzie's Mission involves **BOTH** members of the couple:

- Providing information about themselves
- Watching short videos about genetic carrier screening
- Deciding whether or not to have genetic carrier screening
- Completing at least one online survey
- Providing a mouth swab sample for genetic carrier screening (for those who choose to have screening)

MORE INFORMATION

Phone: 1800 466 466
Email: mackenziesmissionvic@vcgs.org.au
Website: mackenziesmission.org.au

MACKENZIE'S MISSION | Reproductive Genetic Carrier Screening

VCGS leaders in genetic health

TEST REQUEST FORM

P 1800 466 466
E mackenziesmissionvic@vcgs.org.au
W mackenziesmission.org.au

1 PATIENT DETAILS

LAST NAME	GIVEN NAMES	SEX	DATE OF BIRTH	LABORATORY REF
ADDRESS	POSTCODE		MOBILE / PHONE (HOME)	

2 TEST REQUIRED

Mackenzie's Mission Reproductive Genetic Carrier Screening

SAMPLE TYPE

Mouth swab

3 REPRODUCTIVE PARTNER DETAILS

LAST NAME

GIVEN NAMES

SEX

DATE OF BIRTH

PHONE

ADDRESS ☐ SAME AS PATIENT

4 CLINICAL INFORMATION

☐ NOT PREGNANT

☐ PREGNANT GESTATION (WEEKS) ______ EDD ______

☐ DONOR EGG SPERM EMBRYO (PLEASE CIRCLE)

5 IS THERE A FAMILY HISTORY OF A GENETIC CONDITION?

PATIENT ☐ NO ☐ YES Specify: ______

REPRODUCTIVE PARTNER ☐ NO ☐ YES Specify: ______

6 SPECIMEN COLLECTION

PATIENT

TIME OF COLLECTION ______

DATE OF COLLECTION ______

REPRODUCTIVE PARTNER

TIME OF COLLECTION ______

DATE OF COLLECTION ______

7 HEALTHCARE PROVIDER'S SIGNATURE AND REQUEST DATE

DATE ______

SIGNATURE ______

☐ **THIS IS A SAMPLE RECOLLECT**

SIGNATURE/IDENTIFICATION OF COLLECTOR (REQUIRED FOR BLOOD SAMPLES ONLY)

______

REQUESTING HEALTHCARE PROVIDER (PROVIDER #, INITIALS AND ADDRESS)

UNIQUE ACCESS CODE: 1-A11-111

IF YOU AND YOUR REPRODUCTIVE PARTNER CHOOSE TO HAVE SCREENING, THIS FORM MUST BE SENT WITH YOUR SAMPLES FOR TESTING

MACKENZIE'S MISSION
REPRODUCTIVE GENETIC CARRIER SCREENING

PLEASE KEEP FOR YOUR RECORDS

mackenziesmission.org.au

STUDY PARTNERS: murdoch children's research institute; VCGS leaders in genetic health; Australian Genomics

VERSION 1.0 JAN 2020
