# Supplementary material for: The Australian Reproductive Genetic Carrier Screening Project (Mackenzie’s Mission): Design and Implementation
Source: J Pers Med. 2022 Oct 28;12(11):1781. doi: 10.3390/jpm12111781 (PMC9698511; doi:10.3390/jpm12111781)
Supplement: Supplementary file 1 [file jpm-12-01781-s001.zip › Supplementary File S3 - MM gene and condition list_V2.2_23.07.2021.pdf]

MACKENZIE'S MISSION
REPRODUCTIVE GENETIC CARRIER SCREENING

List of genes and conditions screened in Mackenzie's Mission

Please note that some genes appear on this list more than once, as changes in some genes can cause more than one different condition.

Condition	Genes
**Syndromes with intellectual disability**	
**Multiple congenital abnormalities with intellectual disability**	
Achalasia-addisonianism-alacrimia syndrome	AAAS
Al Kaissi syndrome	CDK10
Athabaskan brainstem dysgenesis syndrome	HOXA1
Arthrogryposis, intellectual disability, and seizure disorder	SLC35A3
3MC syndrome	COLEC11, MASP1
Bardet-Biedl syndrome	ARL6, BBS1, BBS10, BBS12, BBS2, BBS4, BBS5, BBS7, BBS9, LZTFL1, MKKS, MKS1, SDCCAG8, TTC8
Basel-Vanagait-Smirin-Yosef syndrome	MED25
Behr syndrome	OPA1
Boucher-Neuhauser syndrome	PNPLA6
Bosley-Salih-Alorainy syndrome	HOXA1
Brunner syndrome	MAOA
Goldberg-Shprintzen megacolon syndrome	KIFBP
Borjeson-Forssman-Lehmann syndrome	PHF6
Bloom syndrome	BLM
Partington syndrome	ARX
Pitt-Hopkins-like syndrome	CNTNAP2
Polyhydramnios, megalencephaly, and symptomatic epilepsy	STRADA
PERCHING syndrome	KLHL7
Shaheen syndrome	COG6
Growth retardation, intellectual developmental disorder, hypotonia, and hepatopathy	IARS1
Cataracts, growth hormone deficiency, sensory neuropathy, sensorineural hearing loss, and skeletal dysplasia (CAGSSS)	IARS2
Carey-Fineman-Ziter syndrome	MYMK
Cerebellofaciodental syndrome	BRF1
Craniofacial dysmorphism, skeletal anomalies, and intellectual disability syndrome	TMCO1

CHIME syndrome	PIGL
COACH syndrome	CC2D2A, RPGRIP1L, TMEM67
Cockayne syndrome	ERCC4, ERCC5, ERCC6, ERCC8
Cohen syndrome	VPS13B
Cerebrooculofacioskeletal syndrome (COFS)	ERCC2, ERCC6
Coffin-Lowry syndrome	RPS6KA3
Cowchock syndrome	AIFM1
De Sanctis-Cacchione syndrome	ERCC6
Developmental delay with short stature, dysmorphic features, and sparse hair	DPH1
Donnai-Barrow syndrome	LRP2
DOOR syndrome	TBC1D24
XFE progeroid syndrome	ERCC4
Desmosterolosis	DHCR24
Dyggve-Melchior-Clausen disease	DYM
Elsahy-Waters syndrome	CDH11
Fragile X syndrome	FMR1
Frontometaphyseal dysplasia	FLNA
Galloway-Mowat syndrome	WDR73, OSGEP
Gillespie syndrome	ITPR1
Griscelli syndrome	RAB27A
HSAN2D syndrome	SCN9A
Hypoparathyroidism-retardation-dysmorphism syndrome	TBCE
Hypotonia, infantile, with psychomotor retardation and characteristic facies	TBCK, UNC80, NALCN
Jawad syndrome	RBBP8
Jensen syndrome	TIMM8A
Johanson-Blizzard syndrome	UBR1
IFAP syndrome with or without BRESHECK syndrome	MBTPS2
Immunoskeletal dysplasia with neurodevelopmental abnormalities	EXTL3
Infantile liver failure syndrome	LARS1
Intellectual developmental disorder with dysmorphic facies, seizures, and distal limb anomalies	OTUD6B
Intellectual developmental disorder with cardiac arrhythmia	GNB5
Kohlschutter-tonz syndrome	ROGDI
Lujan-Fryns syndrome	MED12
Ohdo syndrome	MED12

Opitz-Kaveggia syndrome	MED12
Opitz GBBB syndrome	MID1
Oliver-McFarlane syndrome	PNPLA6
Mosaic variegated aneuploidy syndrome	BUB1B
MEHMO syndrome	EIF2S3
Muscular dystrophy, congenital, with cataracts and intellectual disability	INPP5K
Nijmegen breakage syndrome	NBN, RAD50
Nance-Horan syndrome	NHS
Neurodevelopmental disorder with brain anomalies and additional features	PLAA, PRUNE1, VARS1, WDR45B
Multiple congenital anomalies-hypotonia-seizures syndrome	PIGA, PIGN, PIGT
Renpenning syndrome	PQBP1
Salt and pepper developmental regression syndrome	ST3GAL5
Seckel syndrome	ATR, CENPJ, CEP152, RBBP8
SESAME syndrome	KCNJ10
Smith-Lemli-Opitz syndrome	DHCR7
Spastic paraplegia and psychomotor retardation with or without seizures	HACE1
LIG4 syndrome	LIG4
Wieacker-Wolff syndrome	ZC4H2
Alacrima, achalasia, and intellectual disability syndrome	GMPPA
Chudley-McCullough syndrome	GPSM2
Growth retardation, developmental delay, coarse facies, and early death	FTO
Martsolf syndrome	RAB3GAP2
Pierson syndrome	LAMB2
Hemorrhagic destruction of the brain with subependymal calcification and cataracts	JAM3
Hennekam lymphangiectasia-lymphedema syndrome	CCBE1, FAT4
Perlman syndrome	DIS3L2
Temtamy preaxial brachydactyly syndrome	CHSY1
Filippi syndrome	CKAP2L
Fraser syndrome	FRAS1, FREM2
Orofaciodigital syndrome	CPLANE1, C2CD3, DDX59, SERPINH1, TMEM107, TCTN3
Roberts syndrome	ESCO2

SC phocomelia syndrome	ESCO2
Warburg micro syndrome	RAB18, RAB3GAP1, RAB3GAP2
Woodhouse-Sakati syndrome	DCAF17
Van Maldergem syndrome	DCHS1, FAT4
Warsaw breakage syndrome	DDX11
You-Hoover-Fong syndrome	TELO2
**Syndromic microcephaly**	
Microcephaly, epilepsy, and diabetes syndrome	IER3IP1
Microcephaly, progressive, seizures, and cerebral and cerebellar atrophy	QARS1
Microcephaly-capillary malformation syndrome	STAMBP
Microcephaly, short stature, and impaired glucose metabolism	TRMT10A
Microcephaly, short-stature and endocrine dysfunction	XRCC4
Microcephaly, short stature, and limb abnormalities	DONSON
Microcephaly and chorioretinopathy	TUBGCP4, TUBGCP6
Microcephaly, seizures, spasticity, and brain calcification	PCDH12
**X-linked syndromic intellectual disability**	
Turner type	HUWE1
Claes-Jensen type	KDM5C
Christianson type	SLC9A6
Siderius type	PHF8
Type 14	UPF3B
CK syndrome	NSDHL
Snyder-Robinson type	SMS
Nascimento type	UBE2A
Raymond type	ZDHHC9
Intellectual disability, truncal obesity, retinal dystrophy, and micropenis	INPP5E
Intellectual disability, X-linked, with cerebellar hypoplasia and distinctive facial appearance	OPHN1
**Syndromic brain malformations**	
MASA syndrome	L1CAM
CRASH syndrome	L1CAM
Agenesis of the corpus callosum with peripheral neuropathy (Andermann syndrome)	SLC12A6
Acrocallosal syndrome	KIF7

Proud syndrome	ARX
Temtamy syndrome	C12orf57
Cerebroretinal microangiopathy with calcifications and cysts	CTC1
Vici syndrome	EPG5
Proliferative vasculopathy and hydraencephaly-hydrocephaly syndrome	FLVCR2
Neurodevelopmental disorder and structural brain anomalies with or without seizures and spasticity	PTPN23 **Not screened in WA, QLD and SA until 08/02/2022*
**Syndromic skin conditions with intellectual disability**	
Cerebral dysgenesis, neuropathy, ichthyosis, and palmoplantar keratoderma syndrome	SNAP29
Adams-Oliver syndrome	DOCK6, EOGT
**Syndromic vision conditions with intellectual disability**	
Peter's plus syndrome	B3GLCT
Congenital cataracts, hearing loss, and neurodegeneration	SLC33A1
Knobloch syndrome	COL18A1
Lowe syndrome	OCRL
Kaufman oculocerebrofacial syndrome	UBE3B
Kahrizi syndrome	SRD5A3
Optic atrophy with or without ataxia, intellectual disability, and seizures	RTN4IP1
Norrie disease	NDP
**Syndromic growth conditions with intellectual disability**	
Simpson-Golabi-Behmel syndrome	OFD1, GPC3
**Severe, lethal, neonatal syndromes**	
Meckel syndrome	CC2D2A, CEP290, MKS1, NPHP3, RPGRIP1L, TMEM216, TMEM231, TMEM67
Alkuraya-Kucinskas syndrome	KIAA1109
Bowen-Conradi syndrome	EMG1
Fetal akinesia deformation sequence	RAPSN
Lethal congenital contracture syndrome	CNTNAP1, GLE1, GLDN
Ventriculomegaly with cystic kidney disease	CRB2
Hydrolethalus syndrome	HYLS1, KIF7
TARP syndrome	RBM10
Rigidity and multifocal seizure syndrome, lethal neonatal	BRAT1

Syndromes without intellectual disability	
**Multiple pterygium syndrome**	
Lethal type	CHRNA1, RIPK4
Escobar syndrome	CHRNG
**Multiple congenital abnormalities**	
Burn-McKeown syndrome	TXNL4A
Bifid nose with or without anorectal and renal anomalies	FREM1
Crisponi syndrome	CRLF1, CLCF1
McKusick-Kaufman syndrome	MKKS
Shwachman-Diamond syndrome	SBDS
Split-hand foot malformation	WNT10B
Werner syndrome	WRN
VACTERL association X-linked	ZIC3
Lipodystrophy, congenital generalized	BSCL2, CAVIN1
Wolfram syndrome	CISD2, WFS1
Urofacial syndrome	HPSE2, LRIG2
**Syndromic skin and skeletal conditions**	
Rothmund-Thomson syndrome	RECQL4
Alstrom syndrome	ALMS1
GAPO syndrome	ANTXR1
HELIX syndrome	CLDN10
Haim-Munk syndrome	CTSC
Laryngoonychocutaneous syndrome	LAMA3
Miller syndrome	DHODH
Macrocephaly, alopecia, cutis laxa, and scoliosis	RIN2
Mandibuloacral dysplasia with type B lipodystrophy	ZMPSTE24
Dyskeratosis congenita	DKC1, RTEL1, WRAP53
Papillon-Lefevre syndrome	CTSC
Spondyloocular syndrome	XYLT2
Treacher-Collins syndrome	POLR1C
Schimke immunoosseous dysplasia	SMARCAL1
**Syndromic vision and hearing conditions**	
Usher syndrome	ADGRV1, CDH23, CLRN1, MYO7A, PCDH15, USH1C, USH1G, USH2A, WHRN
Retinitis pigmentosa with skeletal anomalies	CWC27

Jalili syndrome	CNNM4
**Syndromic vision and renal conditions**	
Senior-Loken syndrome	CEP290, NPHP1, NPHP4, SDCCAG8, IQCB1, WDR19
**Mitochondrial conditions**	
**Conditions affecting multiple body systems**	
Combined oxidative phosphorylation deficiency	AARS2, C12orf65, CARS2, FARS2, ELAC2, GFM1, GTPBP3, MTFMT, MTO1, NARS2, RMND1, TSFM, TUFM, VARS2, TRIT1, EARS2
**Leigh and Leigh-like syndrome**	
Mitochondrial complex I deficiency	ACAD9, FOXRED1, NUBPL, NDUFA1, NDUFAF2, NDUFAF5, NDUFAF6, NDUFA10, NDUFA11, NDUFS6, NDUFS4, NDUFS2, NDUFS7, NDUFS8, NDUFS1, NDUFV1, NDUFV2
Leigh syndrome due to cytochrome c oxidase deficiency	COX15
Leigh syndrome, French Canadian type	LRPPRC
**Other mitochondrial conditions**	
Mitochondrial complex II deficiency	SDHAF1
Mitochondrial complex III deficiency	BCS1L, LYRM7, TTC19, UQCRQ
Mitochondrial complex IV deficiency	COX10, COA8, COX20, SURF1, PET100
Mitochondrial complex V deficiency	TMEM70
Mitochondrial DNA depletion syndrome	DGUOK, FBXL4, MGME1, MPV17, RRM2B, SUCLA2, SUCLG1, TK2, TWNK, TYMP
Mitochondrial recessive ataxia syndrome (includes SANDO and SCAE)	TWNK
Multiple mitochondrial dysfunctions syndrome	BOLA3, IBA57, ISCA2, NFU1
Cardioencephalomyopathy, fatal infantile, due to cytochrome c oxidase deficiency 2	COX15, SCO2
Sideroblastic anaemia with B-cell immunodeficiency, periodic fevers, and developmental delay	TRNT1
Leukoencephalopathy with brain stem and spinal cord involvement and lactate elevation	DARS2
Hyperuricemia, pulmonary hypertension, renal failure, and alkalosis (HUPRA syndrome)	SARS2
HSD10 disease	HSD17B10
Mohr-Tranebjaerg syndrome	TIMM8A

Mitochondrial neurodevelopmental disorder, with abnormal movements and lactic acidosis	WARS2
Myopathy, lactic acidosis, and sideroblastic anaemia	PUS1, LARS2, YARS2
Myopathy, mitochondrial, and ataxia	MSTO1 **Not screened in WA, QLD and SA until 08/02/2022*
Mitochondrial short-chain enoyl-CoA hydratase 1 deficiency	ECHS1
**Lysosomal storage disorders**	
**Mannosidosis**	
Alpha	MAN2B1
Beta	MANBA
**Mucopolysaccharidosis**	
Mucopolysaccharidosis	GALNS, GNS, GUSB, IDS, IDUA
Type VI (Maroteaux-Lamy)	ARSB
Type IVB (Morquio)	GLB1
Type IIIA (Sanfilippo A)	SGSH
Type IIIB (Sanfilippo B)	NAGLU
Type IIIC (Sanfilippo C)	HGSNAT
**Cystinosis**	
Atypical nephropathic	CTNS
Nephropathic	CTNS
Late-onset juvenile or adolescent nephropathic	CTNS
Ocular non-nephropathic	CTNS
**Other lysosomal storage disorders**	
Galactosialidosis	CTSA
Yunis-Varon syndrome	FIG4
Fucosidosis	FUCA1
Farber lipogranulomatosis	ASAH1
Glycogen storage disease (Pompe)	GAA
Geleophysic dysplasia	ADAMTSL2
Krabbe disease	GALC, PSAP
Fabry disease	GLA
GM1-gangliosidosis	GLB1
GM2-gangliosidosis	HEXA, GM2A
Metachromatic leukodystrophy	ARSA, PSAP
Mucolipidosis	GNPTAB, GNPTG, MCOLN1

Polyglucosan body myopathy 1 with or without immunodeficiency	RBCK1
Tay-Sachs disease	HEXA
Sandhoff disease	HEXB
Chediak-Higashi syndrome	LYST
Aspartylglucosaminuria	AGA
Schindler disease	NAGA
Sialidosis	NEU1
Combined SAP deficiency	PSAP
Marinesco-Sjogren syndrome	SIL1
Sialic acid storage disorder	SLC17A5
Niemann-Pick disease	NPC1, NPC2, SMPD1
**Metabolic conditions**	
**Peroxisome biogenesis disorders**	
Including Zellweger syndrome, neonatal adrenoleukodystrophy and infantile Refsum disease	PEX1, PEX10, PEX11B, PEX12, PEX13, PEX16, PEX2, PEX26, PEX3, PEX5, PEX6, PEX7
**Organic acidemias**	
Argininosuccinic aciduria	ASL
3-methylglutaconic aciduria	AUH, CLPB, DNAJC19, HTRA2, OPA3, SERAC1
D-2-hydroxyglutaric aciduria	D2HGDH
Glutaricaciduria	GCDH
D-glyceric aciduria	GLYCTK
L-2-hydroxyglutaric aciduria	L2HGDH
Methylmalonic aciduria	MMADHC, MMUT
Methylmalonic aciduria and homocystinuria	LMBRD1, MMACHC, MMADHC
Alpha-methylacetoacetic aciduria	ACAT1
Methylmalonic aciduria, vitamin B12-responsive	MMAA, MMAB
Mevalonic aciduria	MVK
Combined D-2- and L-2-hydroxyglutaric aciduria	SLC25A1
Isovaleric acidemia	IVD
Glutaric acidemia	ETFA, ETFB, ETFDH
**Other metabolic conditions**	
Adenylosuccinase deficiency	ADSL
Arts syndrome	PRPS1
Chanarin-Dorfman syndrome	ABHD5

Galactosemia	GALT **Not screened in WA, QLD and SA until 08/02/2022*
Glycogen storage disease	AGL, G6PC, GYS2, GBE1, LDHA, PFKM, SLC37A4
GABA-transaminase deficiency	ABAT
Fanconi-Bickel syndrome	SLC2A2
Hyperinsulinemic hypoglycemia	ABCC8, HADH, KCNJ11
Hyperoxaluria	AGXT
Hypermanganesemia with dystonia	SLC39A14
Succinic semialdehyde dehydrogenase deficiency	ALDH5A1
Fructose intolerance	ALDOB
Congenital disorders of glycosylation	ALG1, ALG11, ALG12, ALG3, ALG6, ALG8, ALG9, CCDC115, COG6, COG7, DOLK, DPAGT1, MGAT2, MPI, PGM1, PMM2, RFT1, SLC39A8, SSR4, SRD5A3, TMEM165
Congenital disorder of deglycosylation	NGLY1
Glycine encephalopathy	AMT, GLDC
Glycosylphosphatidylinositol biosynthesis defect	GPAA1
Argininemia	ARG1
Asparagine synthetase deficiency	ASNS
Canavan disease	ASPA
Citrullinemia	ASS1, SLC25A13
Chylomicron retention disease	SAR1B
Menkes disease and occipital horn syndrome	ATP7A
Maple syrup urine disease	BCKDHA, BCKDHB, DBT
Branched-chain ketoacid dehydrogenase kinase deficiency	BCKDK
GRACILE syndrome	BCS1L
Homocystinuria	MMADHC, MTHFR, MTR, MTRR
Lysinuric protein intolerance	SLC7A7
Proteinuria	CLCN5
Prolidase deficiency	PEPD
Hypomagnesemia	CLDN19, SLC30A10, TRPM6
Coenzyme Q10 deficiency	COQ2, COQ4, COQ6, COQ8A
Carbamoylphosphate synthetase I deficiency	CPS1
CPT 2 deficiency	CPT1A, CPT2
Methemoglobinemia	CYB5R3

Metabolic encephalomyopathic crises, recurrent, with rhabdomyolysis, cardiac arrhythmias, and neurodegeneration	TANGO2
Lipid storage myopathy due to flavin adenine dinucleotide synthetase deficiency	FLAD1
Medium-chain acyl-CoA dehydrogenase (MCAD) deficiency	ACADM **Not screened in WA, QLD and SA until 08/02/2022*
Peroxisomal acyl-CoA oxidase deficiency	ACOX1
17-alpha-hydroxylase deficiency	CYP17A1
17,20-lyase deficiency	CYP17A1
Cerebrotendinous xanthomatosis	CYP27A1
Aromatic L-amino acid decarboxylase deficiency	DDC
Dihydrolipoamide dehydrogenase deficiency	DLD
Wolcott-Rallison syndrome	EIF2AK3
Hypophosphatemic rickets	ENPP1
Hyperphosphatasia with intellectual disability syndrome	PIGV, PIGO, PGAP2, PGAP3
Ethylmalonic encephalopathy	ETHE1
Tyrosinemia	FAH, HPD
Fructose-1,6-bisphosphatase deficiency	FBP1
Fumarase deficiency	FH
Cerebral creatine deficiency syndrome	GAMT, GATM, SLC6A8
Gaucher disease	GBA, PSAP
Molybdenum cofactor deficiency	GPHN, MOCS1, MOCS2
Glutathione synthetase deficiency	GSS
3-hydroxyacyl-CoA dehydrogenase deficiency	HADH
LCHAD deficiency	HADHA
Trifunctional protein deficiency	HADHA, HADHB
Hemochromatosis	HAMP, HJV
3-hydroxyisobutryl-CoA hydrolase deficiency	HIBCH
Holocarboxylase synthetase deficiency	HLCS
HMG-CoA lyase deficiency	HMGCL
HMG-CoA synthase-2 deficiency	HMGCS2
Lesch-Nyhan syndrome	HPRT1
D-bifunctional protein deficiency	HSD17B4
Leprechaunism	INSR
Norum disease	LCAT
Lactate dehydrogenase-B deficiency	LDHB
Familial hypercholesterolemia	LDLR, LDLRAP1

Pyruvate dehydrogenase lipoic acid synthetase deficiency	LIAS
Cholesteryl ester storage disease	LIPA
Wolman disease	LIPA
Lipoyltransferase 1 deficiency	LIPT1
Lipoprotein lipase deficiency	LPL
Malonyl-CoA decarboxylase deficiency	MLYCD
Abetalipoproteinemia	MTTP
N-acetylglutamate synthase deficiency	NAGS
N-terminal acetyltransferase deficiency	NAA10
Ornithine transcarbamylase deficiency	OTC
Phenylketonuria (PKU)	PAH
Pyruvate carboxylase deficiency	PC
Hyperphenylalaninemia	PTS, QDPR, DNAJC12
Propionicacidemia	PCCA, PCCB
Proprotein convertase 1 deficiency	PCSK1
Pyruvate dehydrogenase deficiency	PDHA1, PDHB, PDP1
Phosphoglycerate kinase 1 deficiency	PGK1
Phosphoglycerate dehydrogenase deficiency	PHGDH
Refsum disease	PHYH
Pyruvate kinase deficiency	PKLR
Plasminogen deficiency	PLG
Dysplasminogenemia	PLG
Pyridoxamine 5'-phosphate oxidase deficiency	PNPO
Phosphoribosylpyrophosphate synthetase superactivity	PRPS1
Phosphoserine phosphatase deficiency	PSPH
Neu-Laxova syndrome	PHGDH, PSAT1
Riboflavin transport deficiency syndrome	SLC52A2, SLC52A3
Lathosterolosis	SC5D
Emphysema-cirrhosis, due to AAT deficiency	SERPINA1
Hemorrhagic diathesis due to antithrombin Pittsburgh	SERPINA1
Monocarboxylate transporter 1 deficiency	SLC16A1
Thiamine metabolism dysfunction syndrome	SLC19A2, SLC19A3, SLC25A19, TPK1
Carnitine deficiency	SLC22A5
Hyperornithinemia-hyperammonemia-homocitrullinemia syndrome	SLC25A15
Acrodermatitis enteropathica	SLC39A4
Multiple sulfatase deficiency	SUMF1

Salla disease	SLC17A5
Sjogren-Larsson syndrome	ALDH3A2
Sulfite oxidase deficiency	SUOX
Transaldolase deficiency	TALDO1
Barth syndrome	TAZ
Adrenocorticotropic hormone deficiency	TBX19
Transcobalamin II deficiency	TCN2
Hemolytic anaemia due to triosephosphate isomerase deficiency	TPI1
Crigler-Najjar syndrome	UGT1A1
Orotic aciduria	UMPS
VLCAD deficiency	ACADVL
Wilson disease	ATP7B
**Endocrine conditions**	
**Congenital adrenal hyperplasia***	
Severe salt wasting type	CYP11A1, CYP11B2, NR0B1, POU1F1, PROP1, HSD3B2
Lipoid type	STAR
**Excludes 21-hydroxylase deficiency, as the CYP21A2 gene is not screened for technical reasons*	
**Diabetes mellitus**	
Neonatal, with congenital hypothyroidism	GLIS3
Insulin-resistant, with acanthosis nigricans	INSR
**Other endocrine conditions**	
Disordered steroidogenesis due to cytochrome P450 oxidoreductase	POR
Glucocorticoid deficiency	MC2R, MRAP, NNT
Growth hormone deficiency with pituitary anomalies	HESX1
Hyperparathyroidism, neonatal severe	CASR
Hypothryoidism, congenital	TSHB
Insulin-like growth factor resistance	IGF1R
Laron syndrome	GHR
Obesity, morbid, due to leptin deficiency	LEP
Pituitary hormone deficiency	HESX1, LHX3
Proopiomelanocortin (POMC) deficiency	POMC
Rabson-Mendenhall syndrome	INSR

Neurological conditions	
**White matter disorders**	
Adrenoleukodystrophy	ABCD1
Aicardi-Goutieres syndrome	ADAR, RNASEH2A, RNASEH2B, RNASEH2C, SAMHD1, TREX1
Leukodystrophy, hypomyelinating	AIMP1, FAM126A, GJC2, HSPD1, POLR3A, POLR3B, PYCR2, RARS1, UFM1, VPS11
Leukoencephalopathy with ataxia	CLCN2
Leukoencephalopathy with vanishing white matter	EIF2B1, EIF2B2, EIF2B3, EIF2B4, EIF2B5
Leukoencephalopathy, cystic, without megalencephaly	RNASET2
Megalencephalic leukoencephalopathy with subcortical cysts	HEPACAM, MLC1
Hypomyelination with brainstem and spinal cord involvement and leg spasticity (HBSL)	DARS1
Pelizaeus-Merzbacher disease	PLP1
**Congenital brain malformations**	
Pontocerebellar hypoplasia	AMPD2, CLP1, EXOSC3, EXOSC8, RARS2, SEPSECS, TBC1D23, TOE1, TSEN2, TSEN54, VPS53, VRK1
Lissencephaly	ARX, KATNB1, LAMB1, NDE1, DCX, TMTC3
Joubert syndrome	AHI1, ARL13B, CC2D2A, CEP290, CEP41, CPLANE1, CSPP1, INPP5E, KIF7, NPHP1, OFD1, RPGRIP1L, TCTN2, TCTN3, TMEM138, TMEM216, TMEM231, TMEM237, TMEM67
Polymicrogyria	ADGRG1, RTTN
Septooptic dysplasia	HESX1
Band heterotopia	DCX, EML1
Band-like calcification with simplified gyration and polymicrogyria	OCLN
Cerebellar hypoplasia and intellectual disability with or without quadrupedal locomotion	VLDLR
Periventricular heterotopia with microcephaly	ARFGEF2
Poretti-Boltshauser syndrome	LAMA1
Cortical malformations, occipital	LAMC3
**Microcephaly**	
Isolated	ASPM, CDK5RAP2, CENPJ, CEP152, CIT, KIF14, KNL1, MCPH1, MFSD2A, MED17, PNKP, SLC25A19, STIL, WDR62, ZNF335

Hydrocephalus	
Non-syndromic hydrocephalus	L1CAM, CCDC88C, MPDZ
Hydrocephalus with congenital idiopathic intestinal pseudoobstruction	L1CAM
Hydrocephalus due to aqueductal stenosis	L1CAM
Hydrocephalus with Hirschsprung disease	L1CAM
**Neurodegenerative conditions**	
Neuronal ceroid lipofuscinoses	CLN3, CLN5, CLN6, CLN8, CTSD, CTSF, MFSD8, PPT1, TPP1
Parkinson disease, juvenile-onset	DNAJC6, FBXO7, PLA2G6, ATP13A2
Encephalopathy, progressive	BSCL2, TBCD, NAXE
Moyamoya disease	GUCY1A1
Neurodegeneration with brain iron accumulation	C19orf12, PANK2, PLA2G6
Neurodegeneration due to cerebral folate transport deficiency	FOLR1
Neurodegeneration with ataxia, dystonia, and gaze palsy, childhood-onset	SQSTM1
Neurodegeneration, stress-induced, with variable ataxia and seizures	ADPRS **Not screened in WA, QLD and SA until 08/02/2022*
Infantile or childhood-onset striatonigral degeneration	NUP62, VAC14* **Not screened in WA, QLD and SA until 08/02/2022*
PEHO syndrome	ZNHIT3
Infantile cerebellar-retinal degeneration	ACO2
Infantile neuroaxonal dystrophy 1	PLA2G6
Spastic tetraplegia, thin corpus callosum, and progressive microcephaly	SLC1A4
Troyer syndrome	SPART
**Ataxias**	
Ataxia-telangiectasia	ATM, MRE11
Ataxia, early-onset, with oculomotor apraxia and hypoalbuminemia	APTX
Ataxia, cerebellar, Cayman type	ATCAY
Ataxia, posterior column, with retinitis pigmentosa	FLVCR1
Ataxia-oculomotor apraxia 4	PNKP
Ataxia with isolated vitamin E deficiency	TTPA
Cerebellar ataxia, cognitive disability, and disequilibrium (CAMRQ)	WDR81, ATP8A2
Spastic ataxia	KIF1C, MARS2, NKX6-2, SACS

Spinocerebellar ataxia	GRM1, PMPCA, SETX, SNX14, STUB1, SCYL1, TPP1, WWOX
**Movement disorders**	
Choreoacanthocytosis	VPS13A
Dystonia	COL6A3, PRKRA* **Not screened in WA, QLD and SA until 08/02/2022*
Dystonia, dopa-responsive, due to sepiapterin reductase deficiency	SPR
Dystonia, DOPA-responsive, with or without hyperphenylalaninemia	GCH1
Parkinsonism-dystonia, infantile	SLC6A3
Segawa syndrome	TH
**Epilepsy**	
Epilepsy, pyridoxine-dependent	ALDH7A1
Epileptic encephalopathy, early infantile	AP3B2, ARV1, ARX, ARHGEF9, DENND5A, FRRS1L, MECP2, SLC13A5, SLC12A5, SLC25A22, TBC1D24, UBA5, WWOX
Epilepsy, progressive myoclonic	CSTB, EPM2A, GOSR2, KCTD7, NHLRC1, PRICKLE1, SCARB2, TBC1D24
Hyperekplexia	ATAD1, SLC6A5
Epilepsy, early-onset, vitamin B6-dependent	PLPBP
Epilepsy, X-linked, with variable learning disabilities and behaviour disorders	SYN1
Epilepsy, hearing loss, and intellectual disability syndrome	SPATA5
Cortical dysplasia-focal epilepsy syndrome	CNTNAP2
Amish infantile epilepsy syndrome	ST3GAL5
**Intellectual disability**	
Non-syndromic intellectual disability, X-linked	AP1S2, ARX, ATRX, BRWD3, CASK, CLCN4, CUL4B, DLG3, FTSJ1, GDI1, HCFC1, IL1RAPL1, IQSEC2, MECP2, NEXMIF, NLGN4X, PAK3, RAB39B, RLIM, SLC16A2, SYP, THOC2, TSPAN7, USP9X, ZNF711
Non-syndromic intellectual disability, autosomal recessive	ADAT3, CC2D1A, ELP2, GPT2, HERC2, KPTN, LINS1, MAN1B1, MBOAT7, MED23, METTL23, NSUN2, PGAP1, PIGG, TRAPPC9, TTI2, TUSC3
Intellectual developmental disorder with microcephaly and short stature	PUS7 **Not screened in WA, QLD and SA until 08/02/2022*

**Other neurological conditions**	
Sensorineural hearing loss, premature ovarian failure (females), variable intellectual disability, spasticity, ataxia	CLPP
**Cutaneous conditions**	
**Ichthyosis**	
Ichthyosis, congenital, autosomal recessive	ABCA12, ALOX12B, ALOXE3, CERS3, CYP4F22, NIPAL4, TGM1
Ichthyosis, leukocyte vacuoles, alopecia, and sclerosing cholangitis	CLDN1
Epidermolytic hyperkeratosis	KRT10
**Cutis laxa**	
Cutis laxa, autosomal recessive	ALDH18A1, ATP6V0A2, EFEMP2, FBLN5, LTBP4, PYCR1
**Ectodermal dysplasia**	
Ectodermal dysplasia, ectrodactyly and macular dystrophy	CDH3
Ectodermal dysplasia	EDA, EDAR, IKBKG, KRT85
**Cutaneous conditions affecting the nervous system**	
Xeroderma pigmentosum	ERCC2, ERCC4, ERCC5, XPA, XPC
**Other cutaneous conditions**	
Kindler syndrome	FERMT1
Epidermolysis bullosa	COL7A1, COL17A1, DSP, ITGA6, ITGB4, KRT14, KRT5, LAMA3, LAMB3, LAMC2, PLEC
Hyaline fibromatosis syndrome	ANTXR2
Porokeratosis 3, disseminated superficial actinic	MVK
Keratosis linearis with ichthyosis congenital and sclerosing keratoderma	POMP
Netherton syndrome	SPINK5
Poikilderma with neutropenia	USB1
Restrictive dermopathy, lethal	LMNA, ZMPSTE24
Triochthiodystrophy	ERCC2, GTF2H5, MPLKIP
Transient bullous of the newborn	COL7A1
**Respiratory conditions**	
**Surfactant conditions**	
Surfactant metabolism dysfunction, pulmonary	ABCA3, SFTPB

**Ciliary dyskinesia**	
Ciliary dyskinesia, primary	OCAD2*, CCDC103, CCDC114, CCDC39, CCDC40, CCNO, DNAAF1, DNAAF3, DNAAF4, DNAAF5, DNAAF6^, GAS8, HYDIN, LRRC6, RSPH1, RSPH4A, RSPH9, SPAG1, ZMYND10<br>**Formerly known as ARMC4*<br>*^Formerly known as PIH1D3*
Ciliary dyskinesia, primary, with or without situs inversus	DNAH11, DNAH5, DNAI1, DNAI2
**Other respiratory conditions**	
Cystic fibrosis	CFTR
Pulmonary veno-occlusive disease	EIF2AK4
Interstitial lung and liver disease	MARS1
**Immunological conditions**	
**Chronic granulomatous disease**	
Deficiency of NCF-1	NCF1
Deficiency of NCF-2	NCF2
Deficiency of CYBA	CYBA
X-linked	CYBB
Combined cellular and humoral immune defects with granulomas	RAG1, RAG2
**Complement deficiencies**	
C1q	C1QA, C1QB, C1QC
C3	C3
C5	C5
C6	C6
C7	C7
C8	C8B
Factor D	CFD
Factor H	CFH
Factor I	CFI
**Immunodeficiencies**	
Immunodeficiency	ATP6AP1, CARD11, CD3D, CTPS1, DOCK2, ICOS, IKBKB, IL12RB1, IL17RA LAT, LRBA, MALT1, ORAI1, PGM3, RORC, STIM1, TYK2
Mycobacteriosis	CYBB, IFNGR1, IFNGR2, STAT1
Purine nucleoside phosphorylase deficiency	PNP

Hyper-IgM	CD40, CD40LG
Hyper-IgD syndrome	MVK
Hyper-IgE recurrent infection syndrome	DOCK8
Centromeric instability-facial anomalies syndrome	DNMT3B, ZBTB24
Combined immunodeficiency, moderate	IL2RG
Combined immunodeficiency and megaloblastic anaemia with or without hyperhomocysteinemia	MTHFD1
**Neutropenia**	
Severe, congenital	G6PC3, HAX1, JAGN1, VPS45, WAS
**Severe combined immunodeficiencies**	
Severe combined immunodeficiency	IL2RG
Adenosine deaminase deficiency	ADA
With microcephaly, growth retardation, and sensitivity to ionizing radiation	NHEJ1
Athabascan type	DCLRE1C
B cell-negative	RAG1, RAG2
T-cell negative, B-cell/natural killer cell-positive type	IL7R, JAK3
Reticular dysgenesis	AK2
**Other immunological conditions**	
Agammaglobulinemia	BTK, IGHM
Autoimmune disease, multisystem, with facial dysmorphism	ITCH
Autoinflammation, lipodystrophy, and dermatosis syndrome	PSMB8
Bone marrow failure syndrome	ERCC6L2, DNAJC21
Bare lymphocyte syndrome	CIITA, RFXAP, TAP1
Candidiasis, familial	CARD9
Histiocytosis-lymphadenopathy plus syndrome	SLC29A3
Hemophagocytic lymphohistiocytosis	PRF1, STX11, STXBP2, UNC13D
Hepatic veno-occlusive disease with immunodeficiency	SP110
Interleukin 1 receptor antagonist deficiency	IL1RN
Immunodysregulation, polyendocrinopathy, and enteropathy	FOXP3
Leukocyte adhesion deficiency	FERMT3, ITGB2
Lymphoproliferative syndrome	CD27, ITK, SH2D1A, XIAP
MHC class II deficiency, complementation group B	RFXANK
Natural killer cell and glucocorticoid deficiency with DNA repair defect	MCM4

Platelet abnormalities with eosinophilia and immune-mediated inflammatory disease	ARPC1B
Properdin deficiency	CFP
Pyogenic bacterial infections, recurrent, due to MYD88 deficiency	MYD88
Selective T-cell defect	ZAP70
T-cell immunodeficiency, congenital alopecia, and nail dystrophy	FOXN1
Darsun syndrome	G6PC3
Majeed syndrome	LPIN2
Omenn syndrome	DCLRE1C, RAG1, RAG2
Wiskott-Aldrich syndrome	WAS
**Gastrointestinal conditions**	
**Severe congenital diarrhea**	
With tufting enteropathy, congenital	EPCAM
Secretory chloride, congenital	SLC26A3
Secretory sodium, congenital,	SPINT2, SLC9A3
Protein-losing enteropathy type	DGAT1
**Hepatic conditions**	
Cholestasis, progressive familial intrahepatic	ABCB11, ABCB4, ATP8B1, TJP2
Hepatic lipase deficiency	LIPC
Porphyria	ALAD, UROS
Liver failure, transient infantile	TRMU
Hypercholanaemia	TJP2
**Other gastrointestinal conditions**	
Microvillus inclusion disease	MYO5B
Bile acid synthesis defect, congenital	AKR1D1, CYP7B1, HSD3B7
Congenital short bowel syndrome	CLMP, FLNA
Complement hyperactivation, angiopathic thrombosis, and protein-losing enteropathy	CD55
Meconium ileus	GUCY2C
Mitchell-Riley syndrome	RFX6
Chronic atrial and intestinal dysrhythmia	SGO1
Inflammatory bowel disease, congenital, severe	IL10RA, IL10RB* **Not screened in WA, QLD and SA until 08/02/2022*
Trichohepatoenteric syndrome	SKIV2L, TTC37
Folate malabsorption, hereditary	SLC46A1

Gastrointestinal defects and immunodeficiency syndrome	TTC7A
Hyperbilirubinemia, familial transient neonatal	UGT1A1
**Haematological conditions**	
**Anaemia**	
Sideroblastic, with ataxia	ABCB7
Anaemia, sideroblastic, pyridoxine-refractory	SLC25A38
Dyserythropoietic anaemia	SEC23B
Haemolytic anaemia due to hexokinase deficiency	HK1
Fanconi anaemia	ERCC4, FANCA, FANCB, FANCC, FANCD2, FANCE, FANCF, FANCG, FANCI, FANCL, UBE2T
**Clotting conditions**	
Hypoprothrombinemia	F2
Factor V deficiency	F5
Factor VII deficiency	F7
Haemophilia A	F8
Haemophilia B	F9
Afibrinogenemia<br>Dysfibrinogenemia<br>Hypodysfibrinogenemia<br>Hypofibrinogenemia	FGA, FGB, FGG
Combined factor V and VIII deficiency	LMAN1, MCFD2
Thrombotic thrombocytopenic purpura	ADAMTS13
Thrombocytopenia, congenital amegakaryocytic	MPL
Thrombophilia	PROC, PROS1
von Willebrand disease	VWF
Thrombocytopenia, X-linked	WAS
**Other haematological conditions**	
Vitamin K-dependent clotting factors, combined deficiency of	VKORC1
Beta thalassemia	HBB
Sickle cell disease	HBB
Atransferrinemia	TF
**Cardiovascular conditions**	
**Arrhythmias**	
Ventricular tachycardia, catecholaminergic polymorphic	CASQ2

Jervell and Lange-Nielsen syndrome	KCNQ1
Ventricular tachycardia, catecholaminergic polymorphic with or without muscle weakness	TRDN
**Cardiomyopathies**	
Cardiomyopathy, dilated, with woolly hair and keratoderma (Naxos disease)	DSP, JUP
Dilated cardiomyopathy	FKTN
**Structural cardiovascular conditions**	
Arterial calcification of infancy	ENPP1
Cardiac valvular dysplasia, X-linked	FLNA
Right atrial isomerism	GDF1
Hypoplastic left heart syndrome	GJA1
Arterial tortuosity syndrome	SLC2A10
Heterotaxy, visceral	ZIC3, MMP21
Congenital heart defects	ZIC3
**Other cardiovascular conditions**	
Sudden cardiac failure, infantile	PPA2
**Renal conditions**	
**Syndromic renal conditions**	
Alport syndrome	COL4A3, COL4A4, COL4A5
Dent disease	OCRL, CLCN5
Renal tubular acidosis with other abnormalities	ATP6V1B1, SLC4A4, SLC4A1
Bartter syndrome	BSND, CLCNKB, KCNJ1, SLC12A1
Renal-hepatic-pancreatic dysplasia	NPHP3, NEK8
Polycystic kidney and hepatic disease	PKHD1
Nephrotic syndrome	COQ8B, DGKE, LAMB2, NPHS1, NPHS2, NUP107, NUP93, PLCE1, SGPL1
**Tubular conditions**	
Renal tubular dysgenesis	ACE, AGT, REN
Renal tubular acidosis	ATP6V0A4
**Other renal conditions**	
Focal segmental glomerulosclerosis	CRB2
Pseudohypoaldosteronism	SCNN1A, SCNN1B

Nephronophthisis and related conditions	ANKS6, DCDC2, INVS, MAPKBP1, NPHP1, NPHP3, NPHP4, TMEM67, TTC21B, WDR19
Nephrogenic diabetes insipidus	AQP2
**Neuromuscular conditions**	
**Atrophy**	
Spinal muscular atrophy with progressive myoclonic epilepsy	ASAH1
Spinal muscular atrophy	SMN1, UBA1
Spinal muscular atrophy with congenital bone fractures	ASCC1
**Arthrogryposis**	
Arthrogryposis, distal	ECEL1, PIEZO2
Arthrogryposis lethal with anterior horn cell disease	GLE1
Arthrogryposis, renal dysfunction, and cholestasis	VIPAS39, VPS33B
Arthrogryposis multiplex congenita	LGI4
**Dystrophy**	
Limb-girdle muscular dystrophy	CAPN3, DYSF, PLEC, SGCA, SGCB, SGCD, SGCG, TCAP, TRAPPC11, TRIM32, TTN
Muscular dystrophy-dystroglycanopathy	B3GALNT2, CRPPA, FKRP, FKTN, GMPPB, LARGE1, POMGNT1, POMGNT2, POMK, POMT1, POMT2, RXYLT1
Muscular dystrophy, congenital	CHKB, LAMA2
Ullrich congenital muscular dystrophy	COL6A1, COL6A2, COL6A3
Duchenne muscular dystrophy	DMD **In NSW, most DMD carriers are unable to be detected due to limitations in testing technology*
Becker muscular dystrophy	DMD **As above*
Emery-Dreifuss muscular dystrophy	EMD, FHL1, LMNA
Muscular dystrophy, rigid spine	SELENON
**Myopathy**	
Myopathy, congenital	ACTA1
Nemaline myopathy	ACTA1, CFL2, KLHL40, KLHL41, LMOD3, NEB, TNNT1, TPM3
Myopathy, centronuclear, autosomal recessive	BIN1, SPEG*<br>**Not screened in WA, QLD and SA until 08/02/2022*
Distal myopathy	DYSF
Myopathy with extrapyramidal signs	MICU1

Myopathy, X-linked	FHL1
Myopathy, X-linked, with excessive autophagy	VMA21
Inclusion body myopathy	GNE
Myopathy, areflexia, respiratory distress, and dysphagia, early-onset	MEGF10
Myotubular myopathy, X-linked	MTM1
Minicore myopathy	RYR1
Myopathy, myofibrillar	KY, PYROXD1
Central core disease	RYR1
Myopathy, early-onset, with fatal cardiomyopathy	TTN
CAP myopathy	TPM3
**Myasthenia**	
Myasthenic syndrome	AGRN, ALG2, CHAT, CHRNA1, CHRND, CHRNE, COLQ, DOK7, DPAGT1, GFPT1, IGHMBP2, MUSK, RAPSN, SLC5A7
**Neuropathy**	
Charcot-Marie-Tooth disease	FGD4, FIG4, GDAP1, LMNA, MFN2, MPZ, MTMR2, NDRG1, PRPS1, PRX, SBF2, SH3TC2
Dysautonomia, familial	ELP1
Insensitivity to pain, congenital	SCN9A, NTRK1
Neuromyotonia and axonal neuropathy	HINT1
Neuropathy, hereditary motor and sensory	HK1, IGHMBP2, KIF1A, SLC25A46
Neuropathy, hereditary sensory and autonomic	NGF, PRDM12, RETREG1, WNK1
Giant axonal neuropathy	GAN
**Rhabdomyolysis**	
Myoglobinuria, acute recurrent	LPIN1
**Spasticity**	
Spastic paralysis, infantile onset ascending	ALS2
Juvenile primary lateral sclerosis	ALS2
Spastic paraplegia	AP4M1, AP4B1, AP4S1, ATP13A2, ALDH18A1, B4GALNT1, CYP2U1, CYP7B1, DDHD2, DSTYK, FA2H, FARS2, GBA2, GJC2, KIF1A, NT5C2, PLP1, PNPLA6, SPG11, VPS37A, ZFYVE26

Connective tissue conditions	
**Ehlers-Danlos syndrome (EDS)**	
Ehlers-Danlos syndrome, progeroid type	ADAMTS2, B3GALT6, B4GALT7, PLOD1
Ehlers-Danlos syndrome, musculocontractural type	CHST14
Ehlers-Danlos syndrome with progressive kyphoscoliosis, myopathy, and hearing loss	FKBP14
**Vascular conditions**	
Polyarteritis nodosa, childhood-onset	ADA2
Meester-Loeys syndrome	BGN

Ocular conditions	
**Albinism**	
Hermansky-Pudlak syndrome	HPS1, HPS3, HPS4, HPS5, HPS6
Oculocutaneous albinism	GPR143, LRMDA, OCA2, SLC24A5, SLC45A2, TYR, TYRP1
**Dystrophies**	
Retinal dystrophy, early-onset severe	LRAT, RCBTB1, CFAP410
Macular dystrophy with central cone involvement	MFSD8
Cone-rod dystrophy	AIPL1, C8orf37, CEP78, CNGB3, KCNV2, PDE6C, RPGRIP1, SEMA4A
**Microphthalmia**	
Isolated	ALDH1A3, RAX, VSX2
With coloboma	STRA6, VSX2
Syndromic	STRA6, RARB
**Other ocular conditions**	
Achromatopsia	ATF6, CNGA3, CNGB3, GNAT2
Aphakia	FOXE3
Congenital cataracts	AGK, FYCO1, NHS, TDRD7
Cone-rod synaptic disorder, congenital non-progressive	CABP4
Choroideremia	CHM
Congenital stationary night blindness	GPR179, NYX
Persistent hyperplastic primary vitreous	ATOH7
Macular degeneration (congenital)	CNGB3, RPGR
Leber congenital amaurosis	AIPL1, CEP290, CRB1, GUCY2D, LCA5, LRAT, NMNAT1, RD3, RDH12, RPE65, RPGRIP1, SPATA7, TULP1

Glaucoma (congenital)	CYP1B1
Peters anomaly	CYP1B1
Retinal arterial macroaneurysm with supravalvular pulmonic stenosis	IGFBP7
Retinitis pigmentosa	AGBL5, AIPL1, C8orf37, CRB1, DHDDS, IFT172, LRAT, MERTK, REEP6, RP2, SEMA4A, SPATA7, TULP1, USH2A
Progressive external ophthalmoplegia	POLG
Brittle cornea syndrome	PRDM5
Corneal opacification and other ocular anomalies	PXDN
Gaze palsy, horizontal, with progressive scoliosis	ROBO3
Foveal hypoplasia, with or without optic nerve misrouting and/or anterior segment dysgenesis	SLC38A8
Optic atrophy	TMEM126A
**Skeletal conditions**	
**Dysplasias**	
Spondyloepiphyseal dysplasia with other abnormalities	CHST3, CCN6
Anauxetic dysplasia	POP1, RMRP
Spondyloepimetaphyseal dysplasia	B3GALT6, NANS
Desbuquois dysplasia	CANT1, XYLT1
Schneckenbecken dysplasia	SLC35D1
Short-rib thoracic dysplasia with or without polydactyly	CEP120, DYNC2H1, DYNC2I1*, DYNC2I2^, DYNC2LI1, KIAA0586, TTC21B, WDR35, IFT140, IFT172, IFT80, NEK1<br>**Formerly known as WDR60*<br>*^Formerly known as WDR34*
Spondylometaepiphyseal dysplasia, short limb-hand type	DDR2
Spondylo-megaepiphyseal-metaphyseal dysplasia	NKX3-2
Chondrodysplasia, Grebe type	GDF5
Oculodentodigital dysplasia	GJA1
Smith-McCort dysplasia	DYM, RAB33B
Omodysplasia	GPC6
Dyssegmental dysplasia, Silverman-Handmaker type	HSPG2
Cranioectodermal dysplasia	IFT122
Opsismodysplasia	INPPL1
Otospondylomegaepiphyseal dysplasia	COL11A2
Greenberg skeletal dysplasia	LBR
Cleft lip/palate-ectodermal dysplasia syndrome	NECTIN1

Spondylometaphyseal dysplasia with additional abnormalities	PCYT1A, CFAP410
Chondrodysplasia, Blomstrand type	PTH1R
Metaphyseal dysplasia without hypotrichosis	RMRP
Craniolenticulosutural dysplasia	SEC23A
Langer mesomelic dysplasia	SHOX
De la Chapelle dysplasia	SLC26A2
Diastrophic dysplasia	SLC26A2
Craniofrontonasal dysplasia	EFNB1
Chondrodysplasia punctata, rhizomelic	AGPS, GNPAT, PEX7
Mandibuloacral dysplasia	LMNA
**Acromesomelic dysplasia**	
Hunter-Thompson type	GDF5
Maroteaux type	NPR2
Demirhan type	BMPR1B
**Arthropathies**	
Arthropathy, progressive pseudorheumatoid	CCN6
Cranioosteoarthropathy	HPGD
Hypertrophic osteoarthropathy	HPGD
Multicentric osteolysis, nodulosis, and arthropathy	MMP2
Camptodactyly-arthropathy-coxa vara-pericarditis syndrome	PRG4
**Short stature and dwarfism**	
Multiple joint dislocations, short stature, craniofacial dysmorphism, and congenital heart defects	B3GAT3
Amelogenesis imperfecta and short stature	LTBP3
Microcephalic osteodysplastic primordial dwarfism	PCNT, RNU4ATAC
Short stature, onychodysplasia, facial dysmorphism, and hypotrichosis	POC1A
Short stature, optic nerve atrophy, and Pelger-Huet anomaly	NBAS
Mulibrey nanism	TRIM37
**Other skeletal conditions**	
3-M syndrome	CCDC8, OBSL1, CUL7
Antley-Bixler syndrome	POR
Hypophosphatasia, infantile	ALPL
Diaphanospondylodysostosis	BMPER
Meier-Gorlin syndrome	CDT1, CDC45, ORC1, ORC6

Osteopetrosis, infantile	CA2, CLCN7, OSTM1, TCIRG1, TNFRSF11A, TNFSF11
Fibrochondrogenesis	COL11A1, COL11A2
Osteogenesis imperfecta, recessive type	CRTAP, FKBP10, P3H1, PPIB*, SERPINF1, WNT1 **Not screened in WA, QLD and SA until 08/02/2022*
Pycnodysostosis	CTSK
Spondylocostal dysostosis	DLL3, HES7, MESP2
Ellis-van Creveld syndrome	EVC, EVC2
Raine syndrome	FAM20C
Bruck syndrome	FKBP10, PLOD2
Spondylocarpotarsal synostosis syndrome	FLNB
Brachydactyly	GDF5
Geroderma osteodysplasticum	GORAB
Craniosynostosis	IL11RA
Alazami syndrome	LARP7
Schwartz-Jampel syndrome	HSPG2
Stuve-Wiedemann syndrome/Schwartz-Jampel type 2 syndrome	LIFR
Acheiropody	LMBR1
Cenani-Lenz syndactyly syndrome	LRP4
Sclerosteosis	LRP4, SOST
Osteoporosis-pseudoglioma syndrome	LRP5
Orofacial cleft	NECTIN1
Brachyolmia 4 with mild epiphyseal and metaphyseal change	PAPSS2
Carpenter syndrome	RAB23, MEGF8
Baller-Gerold syndrome	RECQL4
RAPADILINO syndrome	RECQL4
Cartilage-hair hypoplasia	RMRP
Robinow syndrome	ROR2
Van den Ende-Gupta syndrome	SCARF2
Frank-ter Haar syndrome	SH3PXD2B
Achondrogenesis	SLC26A2, TRIP11
Atelosteogenesis	SLC26A2
Van Buchem disease	SOST
Kenny-Caffey syndrome	TBCE
Paget disease of bone	TNFRSF11B

Ulna and fibula, absence of, with severe limb deficiency	WNT7A
Fuhrmann syndrome	WNT7A
CODAS syndrome	LONP1
Keutel syndrome	MGP
Steel syndrome	COL27A1
