# Supplementary material for: The Australian Reproductive Genetic Carrier Screening Project (Mackenzie’s Mission): Design and Implementation
Source: J Pers Med. 2022 Oct 28;12(11):1781. doi: 10.3390/jpm12111781 (PMC9698511; doi:10.3390/jpm12111781)
Supplement: Supplementary file 1 [file jpm-12-01781-s001.zip › Supplementary File S4 - Laboratory methods.pdf]

Supplementary File S4 – Laboratory methods

The three laboratories used different methods for sequencing and for detection of *FMR1* triplet repeat expansions and *SMN1* exon 7 deletion. The approaches used by each laboratory are described below:

New South Wales Health Pathology Randwick Genomics Laboratory, Sydney

- **Massively parallel sequencing**
  Library preparation was performed using an Agilent SureSelect XT Low Input Clinical Research Exome x2 (Agilent CRE v2) kit, with libraries analysed on an Illumina NovaSeq 6000. Typical coverage was >100x read depth, with >95% of targeted regions covered to a depth of at least 15-fold. Alignment and variant calling for the genes analysed were performed using Illumina Dragen for Enrichment Apps in BaseSpace Australia. Variants were annotated, filtered and analysed using Genomics Annotation and Interpretation Application (GAIA) in-house pipeline version 3.

- **Copy number variant (CNV) analysis (NSW)**
  DMD CNVs were screened using three CNV callers: CoNIFER (PMID: 22585873); DECoN (PMID: 28459104) and XHMM (PMID: 23040492) and annotated using the Genomics Annotation and Interpretation Application (GAIA) in-house pipeline. Any identified CNVs were orthogonally validated prior to reporting.

- ***SMN1* exon 7 copy number**
  The *SMN1* exon 7 copy number was detected using AmplideX SMN1 PCR (Asuragen).
- ***FMR1* triplet repeat analysis**
  AmplideX *FMR1* GS-PCR (Asuragen) was used to determine triplet repeat size.

Pathwest Diagnostic Genomics, Perth

- **Massively parallel sequencing**
  Library preparation was performed using an EF Library preparation (TWIST Biosciences) kit, with a custom probe set (TWIST Biosciences). Libraries were sequenced on an Illumina NextSeq 550. Typical coverage was >150x read depth, with >99.5% of targeted regions covered to a depth of at least 20-fold. Alignment and variant calling for the genes analysed were performed using the Illumina Dragen for Enrichment App in BaseSpace Australia. Variants were annotated, filtered and analysed using Alissa Interpret (Agilent) with an in-house filtering pipeline.
- **Copy number variant (CNV) analysis**
  Panel-wide copy number variants were detected using an in-house method (data not shown). Any identified CNVs were orthogonally validated prior to reporting.
- ***SMN1* exon 7 copy number**
  The *SMN1* exon 7 copy number was detected using a SMN1 Taqman qPCR kit (Thermofisher Scientific) on a QuantStudio5 instrument (Thermofisher Scientific).
- ***FMR1* triplet repeat analysis**
  AmplideX *FMR1* GS-PCR (Asuragen) was used to determine triplet repeat size.

Victorian Clinical Genetics Services, Melbourne

1. **Massively parallel sequencing – current method**
   Whole exome sequencing was performed using massively parallel sequencing (TWIST VCGS whole exome capture, Illumina Sequencers). Typical coverage was >50x read depth, with >98% of targeted regions covered to a depth of at least 10x fold. Alignment and variant calling for the genes analysed were performed using Cpipe (Sadedin et al., 2015). Variants were annotated, filtered and analysed using Alissa Interpret (Agilent) with an in-house filtering pipeline.
   <u>Massively parallel sequencing – method prior to 16.11.2020</u>
   Whole exome sequencing was performed using massively parallel sequencing (Agilent SureSelect XT low input CREv2 kit, Illumina Sequencers). Typical coverage was >100 x read depth, with >95% of targeted regions covered to a depth of at least 20x fold. Alignment and variant calling for the genes analysed were performed using Cpipe (1). Variants were annotated, filtered and analysed using Alissa Interpret (Agilent) with an in-house filtering pipeline.
2. **Copy number variant (CNV) analysis**
   DMD CNVs were detected using the Ximmer CNV analysis pipeline (2) utilising four CNV detection tools (XHMM, ExomeDepth, CODEX and an internal tool) and screened using an internal CNV interpretation tool (CXGo). Only DMD CNVs that met quality thresholds were orthogonally validated.
3. ***SMN1* exon 7 copy number**
   The *SMN1* gene copy number was determined using quantitative real time PCR as previously published (Smith et al., 2007).
4. ***FMR1* triplet repeat analysis**
   AmplideX *FMR1* GS-PCR (Asuragen) was used to determine triplet repeat size.

Variant filtering strategy
All laboratories used an agreed approach to variant filtering. Autosomal variants with >1% allele frequency or >2 homozygotes in gnomAD (v2 or v3) (3) were excluded from analysis. X-linked variants with >0.1% allele frequency or >2 hemizygotes in gnomAD were also excluded. Any variants previously classified by at least one submitter as Likely Pathogenic or Pathogenic in ClinVar (4) were retained for manual curation.

AGG interrupt analysis
Female samples with a *FMR1* premutation of between 55 and 69 CGG repeats were sent to the Children's Hospital at Westmead molecular genetics laboratory a NATA accredited diagnostic laboratory, for AGG interrupt analysis. The polymorphic (CGG)n triplet repeat located within the 5'-untranslated region of the first exon of the *FMR1* gene (NM_002024.5) was amplified by a modified Amplidex™ (Asuragen) PCR (as described by Chen, Hadd (5)), in conjunction with an AGG-specific primer assay (Hayward and Usdin, 2017). Fragments were separated on an AB 3730 DNA Analyser.

References

1. Sadedin SP, Dashnow H, James PA, Bahlo M, Bauer DC, Lonie A, et al. Cpipe: a shared variant detection pipeline designed for diagnostic settings. Genome medicine. 2015;7(1):1-10.
2. Sadedin SP, Ellis JA, Masters SL, Oshlack A. Ximmer: a system for improving accuracy and consistency of CNV calling from exome data. Gigascience. 2018;7(10):giy112.
3. gnomAD. https://gnomad.broadinstitute.org/. Accessed 27 July 2022.
4. ClinVar. https://www.ncbi.nlm.nih.gov/clinvar/. Accessed 27 July 2022.
5. Chen L, Hadd A, Sah S, Filipovic-Sadic S, Krosting J, Sekinger E, et al. An information-rich CGG repeat primed PCR that detects the full range of fragile X expanded alleles and minimizes the need for southern blot analysis. The Journal of Molecular Diagnostics. 2010;12(5):589-600.
